# Inhibition of lipid kinase PIKfyve reveals a role for phosphatase Inpp4b in the regulation of PI(3)P-mediated lysosome dynamics through VPS34 activity

**DOI:** 10.1016/j.jbc.2022.102187

**Published:** 2022-06-26

**Authors:** Golam T. Saffi, Cheng An Wang, Emily M. Mangialardi, Jean Vacher, Roberto J. Botelho, Leonardo Salmena

**Affiliations:** 1 Department of Pharmacology & Toxicology, University of Toronto, Toronto, Ontario, Canada; 2 Institut de Recherches Cliniques de Montréal (IRCM), Département de Médecine, Université de Montréal, Montréal, Québec, Canada; 3 Department of Chemistry and Biology, Ryerson University, Toronto, Ontario, Canada; 4 Princess Margaret Cancer Centre, University Health Network, Toronto, Ontario, Canada

**Keywords:** apilimod, Inpp4b, PIKfyve, Vps34, PtdIns(3)P, lysosomes, FBS, fetal bovine serum, IF, immunofluorescence, INPP4B, inositol polyphosphate 4-phosphatase type II, LY, Lucifer yellow, MEF, mouse embryonic fibroblast, PIKfyve, Phosphoinositide Kinase, FYVE-Type Zinc Finger Containing, PtdIns(3)P, phosphatidylinositol-3-monophosphate, PtdIns(3,5)P2, phosphatidylinositol-3,5-bisphosphate, PtdIns, phosphoinositide, TFEB, Transcription Factor EB

## Abstract

Lysosome membranes contain diverse phosphoinositide (PtdIns) lipids that coordinate lysosome function and dynamics. The PtdIns repertoire on lysosomes is tightly regulated by the actions of diverse PtdIns kinases and phosphatases; however, specific roles for PtdIns in lysosomal functions and dynamics are currently unclear and require further investigation. It was previously shown that PIKfyve, a lipid kinase that synthesizes PtdIns(3,5)P_2_ from PtdIns(3)P, controls lysosome “fusion-fission” cycle dynamics, autophagosome turnover, and endocytic cargo delivery. Furthermore, INPP4B, a PtdIns 4-phosphatase that hydrolyzes PtdIns(3,4)P_2_ to form PtdIns(3)P, is emerging as a cancer-associated protein with roles in lysosomal biogenesis and other lysosomal functions. Here, we investigated the consequences of disrupting PIKfyve function in *Inpp4b*-deficient mouse embryonic fibroblasts. Through confocal fluorescence imaging, we observed the formation of massively enlarged lysosomes, accompanied by exacerbated reduction of endocytic trafficking, disrupted lysosome fusion-fission dynamics, and inhibition of autophagy. Finally, HPLC scintillation quantification of ^3^H-*myo-*inositol labeled PtdIns and PtdIns immunofluorescence staining, we observed that lysosomal PtdIns(3)P levels were significantly elevated in *Inpp4b*-deficient cells due to the hyperactivation of phosphatidylinositol 3-kinase catalytic subunit VPS34 enzymatic activity. In conclusion, our study identifies a novel signaling axis that maintains normal lysosomal homeostasis and dynamics, which includes the catalytic functions of Inpp4b, PIKfyve, and VPS34.

Lysosomes are membrane-bound organelles that serve as a cell’s main degradative center (1, 2). Lysosomes are also key sentinels of cellular nutrient concentration and metabolic activity and have a critical role in initiating diverse signal transduction pathways (3, 4, 5). Lysosomal membranes include diverse phosphoinositides (PtdIns), known to control lysosome function, trafficking, permeability, and general homeostasis through their ability to coordinate recruitment of critical proteins (6). PtdIns exist in seven different forms which are defined by the phosphorylation status of the 3, 4, and/or 5 hydroxyl groups of their inositol head, a process controlled by a number of cellular kinases and phosphatases with PtdIns specificity (7, 8).

Of notable importance on lysosomal membranes is phosphatidylinositol-3,5-bisphosphate [PtdIns(3,5)P_2_], generated through 5`-phosphorylation of phosphatidylinositol-3-monophosphate [PtdIns(3)P] by Phosphoinositide Kinase, FYVE-Type Zinc Finger Containing (PIKfyve) (9, 10). Pharmacological or genetic inhibition of PIKfyve and subsequent depletion of PtdIns(3,5)P_2_ has been demonstrated to disrupt lysosomal-related processes including autophagic flux, endocytic and phagocytic cargo delivery to lysosomes, substrate export from lysosome, and calcium release (11, 12, 13, 14). Notably, inhibition of PIKfyve also leads to a dramatic enlargement of lysosomes, a phenomenon explained in part by defective membrane recycling from endosomes and/or lysosomes (9, 15). PtdIns(3,5)P2 depletion and lysosome enlargement in various cell lines can be effectively induced by the pharmacological PIKfyve inhibitor apilimod (16, 17). Specifically, apilimod interacts with the amino acid asparagine (N1939) predicted to be located within the ATP-binding pocket of the catalytic kinase domain of PIKfyve (18). More recently, Choy *et al.* attributed apilimod-induced lysosome enlargement to the disruption of lysosome fusion-fission cycling and/or disruption of “kiss-and-run”, a term which describes a transient membrane fusion event followed by rapid fission event that permits content exchange between lysosomes (11, 17, 19, 20). In their model, PIKfyve inhibition compromises lysosomal fission (the “run” event) and thus promotes lysosome coalescence which results in increased individual lysosome volume and reduced lysosome numbers (17, 21).

Emerging evidence suggests that other PtdIns including PtdIns(3)P, PtdIns(4)P, and PtdIns(3,4)P_2_ may also play important roles in regulating lysosomal homeostasis. For instance, PtdIns(3)P has been implicated in regulating lysosomal positioning and lysosomal mTORC1 activity (22). VPS34, a class III PtdIns 3-kinase, is recruited by active GTP-bound Rab5 and Rab7 in complex with VPS15, toward early and late endosomes. VPS34-induced PtdIns(3)P generation regulates early and late endosomal morphology (23), intraluminal vesicle formation within late endosomes (24), and endosomal assembly of cargo recycling complex (24). Additionally, mTOR regulation of VPS34 activity controls lysosome tubular morphology (25). PtdIns(4)P has demonstrated important functions in lysosomal membrane fusion in late endocytic trafficking (26). PtdIns(3,4)P_2_ was also demonstrated to promote repression of mTORC1 activity and cell growth through functions on lysosomal membranes (27). Notably, an emerging role for Inositol polyphosphate 4-phosphatase type II (INPP4B), a PtdIns phosphatase that dephosphorylates PtdIns(3,4)P_2_ to form PtdIns(3)P, in lysosome homeostasis has been reported in diverse cancer settings (28, 29). Overexpression of INPP4B leads to depleted intracellular PtdIns(3,4)P_2_ and promoted endosomal trafficking of cargo toward lysosomes in breast cancer (28), and our recent study implicates INPP4B in lysosomal biogenesis in leukemia cells (29). Overall, roles for PtdIns(3,4)P_2_ and PtdIns(3)P in lysosomal function and dynamics are currently unclear and require further investigation.

To better understand a role for INPP4B in lysosome homeostasis, we investigated the consequences of PIKfyve inhibition in *Inpp4b*^*+/+*^ and *Inpp4b*^*−/−*^ mouse embryonic fibroblasts (MEFs). Surprisingly, PIKfyve inhibition with apilimod in *Inpp4b*^*−/−*^ fibroblasts leads to the formation of very massively enlarged lysosomes, compared to the typically enlarged lysosomes produced in *Inpp4b*^*+/+*^ fibroblasts. Our results suggest that exacerbated lysosomal enlargement was, at least in part, a result of aberrantly elevated levels of lysosomal PtdIns(3)P generated by increased activity of the Class III PtdIns 3-kinase VPS34 in *Inpp4b*^*−/−*^ cells. Together, these point to a novel role for Inpp4b in suppressing VPS34 activity and the existence of coordinated functions for Inpp4b, PIKfyve, and VPS34 in maintaining normal lysosomal homeostasis, dynamics, and function.

## Results

### PIKfyve inhibition in Inpp4b-null MEF leads to formation of massively enlarged LAMP1^+^ vacuoles

**Figure 1:**
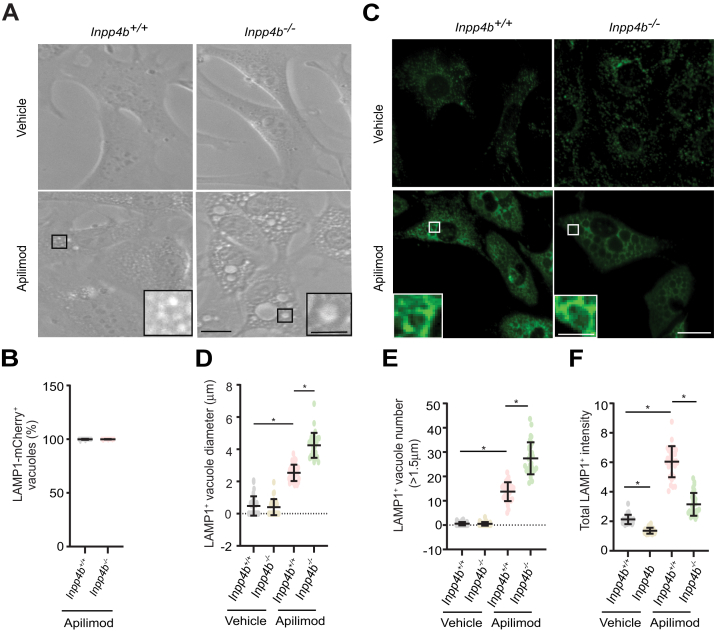
**PIKfyve inhibition in *Inpp4b*-deficient cells leads to altered lysosome homeostasis.***A*, *Inpp4b*^+/+^ or *Inpp4b*^−/−^ MEF cells were incubated with vehicle or 10 nM apilimod for 48 h followed by imaging with DIC optics. *B*, scoring for percentage of LAMP1-mCherry membrane positive vacuoles formed upon apilimod treatment. *C*, fluorescence micrographs of LAMP1 immunostaining for *Inpp4b*^*+/+*^ or *Inpp4b*^*−/−*^ MEFs treated with vehicle or 10 nM apilimod for 48 h. *D*, mean vacuole diameter (μm) positive for LAMP1 membrane immunostain, (*E*) LAMP1 positive vacuole number (>1.5 μm in diameter) per cell across indicated treatments, and (*F*) LAMP1 puncta intensity. The scale bar represents 20 μm, zoomed inset: 5 μm. Data represent ± SD from three independent experiments with 25 to 30 cells assessed per treatment condition per experiment for (*B*–*F*). Statistical significance was measured by ANOVA and multiple Student’s *t* test and represented as ∗ (*p* < 0.05). INPP4B, inositol polyphosphate 4-phosphatase type II; MEF, mouse embryonic fibroblast; PIKfyve, Phosphoinositide Kinase, FYVE-Type Zinc Finger Containing.

Treatment of *Inpp4b*^*+/+*^ and *Inpp4b*^*−/−*^ MEF with the specific PIKfyve inhibitor apilimod generated the formation of enlarged cellular vacuoles at 48 h of apilimod treatment in *Inpp4b*^*+/+*^ MEF by light microscopy; strikingly, *Inpp4b*^*−/−*^ MEF demonstrated an exacerbated vacuolation phenotype with many more massively enlarged vacuoles (Fig. 1*A*). Ectopic expression of the lysosomal specific membrane protein (LAMP1) tagged to mCherry (LAMP1-mCherry) followed by fluorescence microscopy revealed that nearly all the enlarged vacuoles in *Inpp4b*^*+/+*^ and *Inpp4b*^*−/−*^ MEF were membrane positive for LAMP1 indicating a lysosomal origin for enlarged vacuoles (Figs. 1*B* and S1*A*). Quantitation of LAMP1 immunofluorescence (IF) confirmed that enlarged vacuoles were average significantly larger and significantly more abundant in apilimod-treated *Inpp4b*^*−/−*^ MEFs than in *Inpp4b*^*+/+*^ MEF (Fig. 1, *C*–*E*). Total LAMP1 staining demonstrated that vehicle-treated *Inpp4b*^*−/−*^ MEF had less lysosomal content compared to *Inpp4b*^*+/+*^ MEF (Fig. 1*F*). Apilimod treatment significantly increased total LAMP1 staining in both *Inpp4b*^*+/+*^ and *Inpp4b*^*−/−*^ MEF in a proportional manner, indicating that induction of lysosomal content was not compromised in *Inpp4b*^*−/−*^ MEF (Fig. 1, *F*, S1, *E* and *F*). These findings were corroborated with Lysotracker Red (LTR; Fig. S1, *D*–*F*) and ectopic expression of a LAMP1-mCherry in *Inpp4b*^*+/+*^ and *Inpp4b*^*−/−*^ MEF (Fig. S1, *A*–C).

To confirm that the aberrant vacuolation response upon apilimod treatment was indeed a result of *Inpp4b* deficiency, we transduced *Inpp4b*^*−/−*^ MEF with constructs that express *Inpp4b-EGFP*, catalytically dead *Inpp4b*^*C845A*^*-EGFP*, and control *EGFP* and treated with apilimod or vehicle and measured vacuole size and number (Fig. S1, *G*–*I*). *Inpp4b-EGFP* expression in *Inpp4b*^*−/−*^ MEF generated significantly reduced numbers and average size of enlarged vacuoles upon apilimod treatment compared to nontransduced and EGFP negative controls (Fig. S1, *G*–*I*). Notably, transduction of *Inpp4b*^*C845A*^*-EGFP* in *Inpp4b*^*−/−*^ MEF was unable to rescue the number and size of enlarged vacuoles upon apilimod treatment (Fig. S1, G–*I*). Exacerbated lysosomal enlargement observed with INPP4B deficiency was also generalizable to human cells, as determined in U2OS cells upon INPP4B knockdown with RNAi (Fig. S2, *A*–*E*). We also evaluated the effect of Inpp4b suppression on late endosomes. *Inpp4b*^−/−^ MEF displayed reduced CD63^+^ late endosome puncta number (Fig. S3). Notably, apilimod treatment did not alter CD63^+^ late endosome levels, indicating that late endosomes do not reflect the changes observed in lysosomal levels. This suggests that CD63^+^ late endosomes are not affected by apilimod, and that PIKfyve inhibition may selectively disrupt lysosomal homeostasis and dynamics (Fig. S3). Overall, apilimod treatment in *Inpp4b*^*−/−*^ MEF demonstrate an exacerbated lysosomal vacuolation phenotype compared to *Inpp4b*^*+/+*^ MEF that can be rescued by WT Inpp4b, but not a catalytically dead Inpp4b. These data suggest an important role for Inpp4b phosphatase function in maintaining lysosomal dynamics and homeostasis.

### Inpp4b deficiency exacerbates apilimod-mediated inhibition of endocytic trafficking to lysosomes

We attempted to measure lysosome dynamics using Lucifer yellow (LY), a membrane impermeable fluorescent dye that is internalized by endocytosis and accumulates within lysosomes. LY fluorescence provides an effective tool to specifically delineate lysosomes for accurate measurement of lysosome number and volume (30, 31). MEF were treated with vehicle or apilimod prior to LY pulses of 1, 2, and 4 h. Flow cytometry was used to measure LY uptake which demonstrated that apilimod elevated total intracellular LY-fluorescence similarly in both *Inpp4b*^*+/+*^ and Inpp*4b*^*−/−*^ MEF for up to 4 h (Fig. S4). To monitor specific LY trafficking to lysosomes, we labeled lysosomes with ectopic expression of LAMP1-mCherry and measured colocalization with LY (Fig. S5*A*). Apilimod treatment demonstrated a markedly reduced accumulation of LY to lysosomes in *Inpp4b*^*+/+*^ MEF. Comparatively, *Inpp4b*^*−/−*^ MEF has significantly less LY accumulation at lysosomes compared to *Inpp4b*^*+/+*^ MEF for up to 2 h, indicating a slower colocalization of LY with lysosomes with *Inpp4b* deficiency (Fig. S5, *A* and *B*). We performed the same assay but replaced LY with DQ-BSA, bovine serum albumin labeled with a self-quenched BODIPY TR-X dye that upon lysosomal delivery is cleaved by lysosomal hydrolases, resulting in bright green fluorescent signal (32, 33). We observed that DQ-BSA activation was delayed but not inhibited in apilimod-treated Inpp*4b*^*−/−*^ MEF (Fig. S5*C*), suggesting that degradation of lysosomal cargo was not compromised. These findings indicate that PIKfyve inhibition reduces the ability of endocytosed cargo to reach terminal lysosomes, with no major effects on lysosomal proteolysis. Interestingly, like the vacuolation phenotype, apilimod-mediated accumulation was exacerbated in *Inpp4b*^*−/−*^ MEF, further indicating a role for Inpp4b in lysosomal trafficking (11, 34).

### Inpp4b deficiency exacerbates apilimod-mediated lysosome fusion-fission dynamics

**Figure 2:**
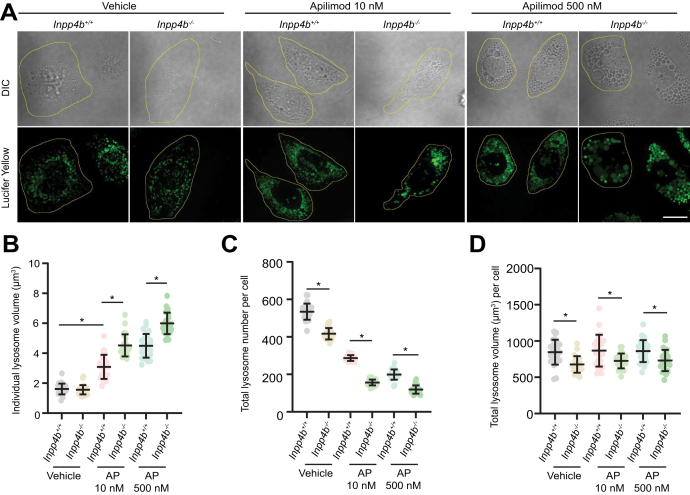
**Lysosome volume and numbers are altered upon PIKfyve inhibition in *Inpp4b*-deficient cells.***A*, lysosomes of *Inpp4b*^*+/+*^ or *Inpp4b*^*−/−*^ MEFs prelabeled with Lucifer *Yellow* and exposed to vehicle or apilimod at indicated concentrations for 1 h. Fluorescence micrographs represent Z-projections of 25 to 30 Z-planes acquired through spinning disc confocal microscopy. Image analysis performed for individual lysosome volume (B), lysosome number per cell (C), and total lysosome volume per cell (D). The scale bar represents 25 μm. Data represent mean ± SD from three independent experiments, with 25 to 30 cells assessed per treatment condition per experiment. Statistical significance was measured by ANOVA and multiple Student’s *t* test and represented as ∗ (*p* < 0.05). INPP4B, inositol polyphosphate 4-phosphatase type II; MEF, mouse embryonic fibroblast; PIKfyve, Phosphoinositide Kinase, FYVE-Type Zinc Finger Containing.

Given that apilimod blocks lysosomal accumulation of endocytic cargo such as LY, we adjusted our treatment conditions so that MEF were first pulsed with LY followed by acute treatment with apilimod (Fig. 2*A*). In these experiments, quantitation of LY-labeled lysosomes revealed that individual basal lysosome size was similar in *Inpp4b*^*+/+*^ or *Inpp4b*^*−/−*^ MEF. Apilimod induced significant lysosomal enlargement compared to vehicle, and lysosome enlargement in *Inpp4b*^*−/−*^ MEF was significantly larger than *Inpp4b*^*+/+*^ MEF (Fig. 2, *A* and *B*). *Inpp4b*^*−/−*^ MEF have fewer total lysosomes than *Inpp4b*^*+/+*^ MEF; and apilimod treatment reduced lysosome numbers in both *Inpp4b*^*+/+*^ or *Inpp4b*^*−/−*^ MEF (Fig. 2, *A* and *C*). Notably, total lysosomal content as measured by total cellular LY accumulation was significantly lower in *Inpp4b*^*−/−*^ MEF and remained unchanged upon apilimod treatment (Fig. 2, *A* and *D*). These data conform to a model whereby apilimod treatment promotes lysosomal coalescence, which is manifested as increased individual lysosome volume and reduced total lysosome numbers (16, 17, 35). Notably, *Inpp4b* deficiency exacerbates apilimod-induced lysosomal enlargement, thus providing further evidence of a role for Inpp4b in lysosomal homeostasis and dynamics which is revealed upon apilimod treatment.

**Figure 3:**
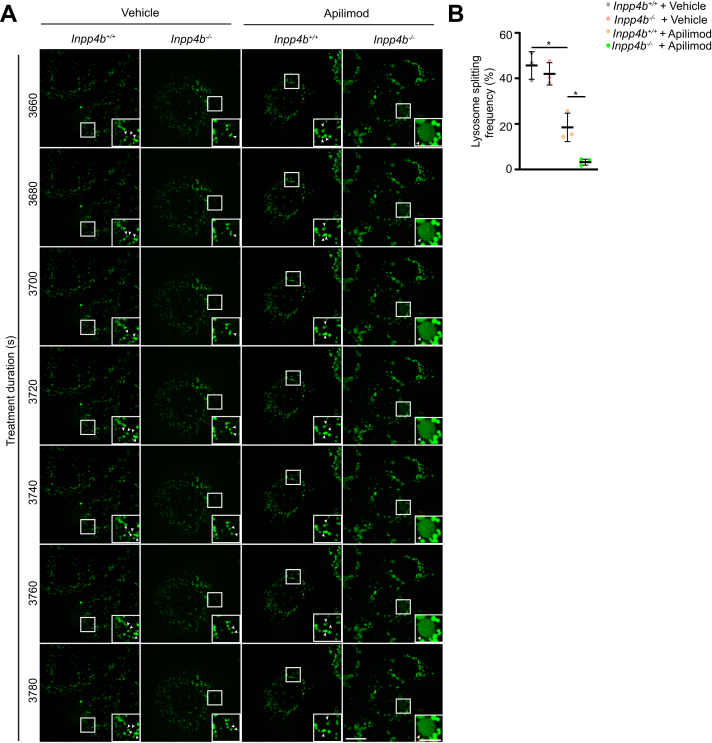
**Lysosome “kiss-and-run” dynamics are disrupted upon PIKfyve inhibition in *Inpp4b*-deficient cells.***A*, *Inpp4b*^*+/+*^ or *Inpp4b*^*−/−*^ MEFs were prelabeled with Lucifer Yellow followed by treatment with vehicle or 10 nM apilimod. Time in sec (s) refers to time post treatment. See Movies S1–S4 for full capture imaging videos. Inset represents an enlarged section from the field of view tracking individual lysosome particle dynamics over time. The scale bar represents 20 μm, zoomed inset: 10 μm. *B*, lysosome splitting frequency over 15 min for WT or Null MEFs treated with vehicle or apilimod demonstrate reduced splitting in PIKfyve inhibited WT MEFs and to a greater extent in PIKfyve inhibited Null MEFs, indicative of possible reduced lysosome fission. Data represent mean ± SD from three independent experiments, where at least a total of 1300 lysosomes were tracked for 1 to 3 cells over 15 min. Statistical significance was measured by ANOVA and multiple Student’s *t* test and represented as ∗ (*p* < 0.05). INPP4B, inositol polyphosphate 4-phosphatase type II; MEF, mouse embryonic fibroblast; PIKfyve, Phosphoinositide Kinase, FYVE-Type Zinc Finger Containing.

Inhibition of lysosomal fission has been proposed as an explanation for apilimod-induced lysosome enlargement (16, 17, 35). To investigate the specific consequence of *Inpp4b* deficiency on “kiss-and-run” and/or “fusion-and-fission” cycles, live-imaging of LY-labeled lysosomes was recorded. *Inpp4b*^*+/+*^ and *Inpp4b*^*−/−*^ MEF treated with either vehicle or apilimod were monitored for up to 15 min and lysosomal fission events were recorded. Despite presenting similar fission rates in vehicle-treated *Inpp4b*^*+/+*^ and *Inpp4b*^*−/−*^ MEF, apilimod significantly reduced lysosome fission rates in *Inpp4b*^*+/+*^ MEF, and remarkably fission was nearly abrogated in *Inpp4b*^*−/−*^ MEF (Fig. 3, *A* and *B* and Movies S1–S4). This suggests that the enhanced lysosomal coalescence observed in *Inpp4b*^*−/−*^ MEF upon apilimod treatment is due to an exacerbated inhibition of lysosomal fission and *Inpp4b* deficiency alters lysosome dynamics such that they are sensitized to the lysosomal fission-inhibiting effects of apilimod.

### Apilimod treatment induces differential effects on gene expression and lysosome function in Inpp4b^+/+^ and Inpp4b^−/−^ MEF

**Figure 4:**
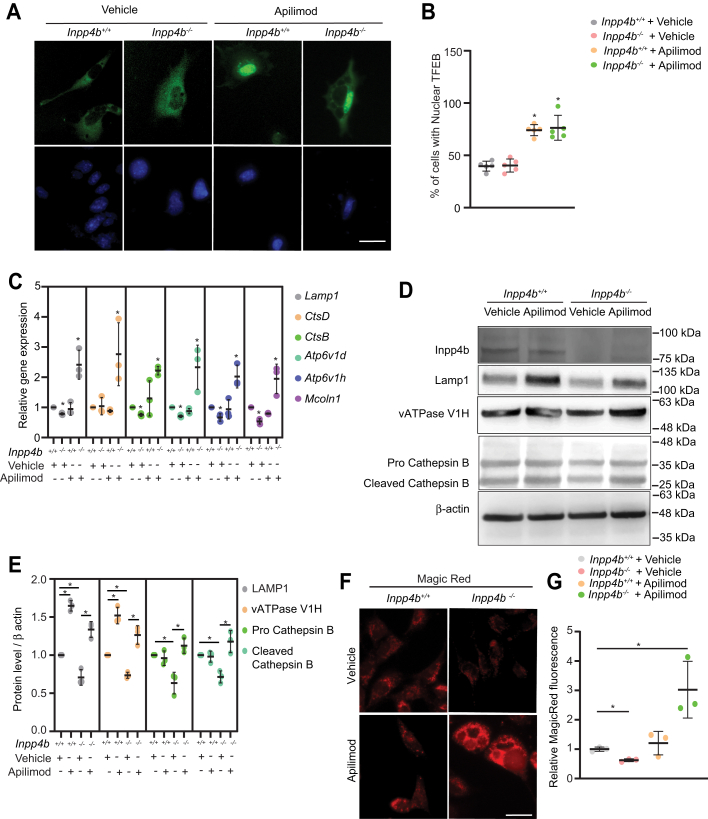
**PIKfyve inhibition in *Inpp4b*-deficient cells disrupts lysosome gene expression.***A*, *Inpp4b*^*+/+*^ or *Inpp4b*^*−/−*^ MEFs transiently expressing *pEGFP-TFEB* and treated with vehicle or apilimod 10 nM for 48 h. DNA was stained with Hoechst 33342. *B*, TFEB nuclear translocation measured as percentage of cells demonstrating nuclear EGFP-TFEB for (*A*). The scale bar represents 25 μm. *C*, *Inpp4b*^*+/+*^ or *Inpp4b*^*−/−*^ MEFs treated with vehicle or apilimod 10 nM for 48 h followed by qRT-PCR analysis of lysosome gene expression normalized against Actb. Shown is mean ± SD from three independent experiments. *D*, immunoblot of *Inpp4b*^*+/+*^ or *Inpp4b*^*−/−*^ MEFs treated with vehicle or apilimod 10 nM for 48 h followed by assessment of select lysosomal proteins as indicated. *E*, analysis of protein levels from (*D*) shown as mean ± SD from three independent experiments. Data represent ± SD from five independent experiments with 30 to 35 cells assessed per treatment condition per experiment for (*A* and *B*). *F*, *Inpp4b*^*+/+*^ or *Inpp4b*^*−/−*^ MEF treated with vehicle or apilimod 10 nM for 48 h followed by Magic red incubation for 1 h. The scale bar represents 25 μm. *G*, quantification of Magic red intensity from (*F*) as measured through flow cytometry and mean ± SD from three independent experiments. Statistical significance was measured by ANOVA and multiple Student’s *t* test and represented as ∗ (*p* < 0.05). INPP4B, inositol polyphosphate 4-phosphatase type II; MEF, mouse embryonic fibroblast; PIKfyve, Phosphoinositide Kinase, FYVE-Type Zinc Finger Containing; TFEB, Transcription Factor EB.

To shed light on the exacerbated vacuolation phenotypes observed with *Inpp4b* deficiency, we assessed the effects of apilimod treatment on lysosomal biogenesis in *Inpp4b*^*+/+*^ and *Inpp4b*^*−/−*^ MEF. Firstly, evaluation of nuclear translocation of Transcription Factor EB (TFEB) (36), a key regulator of lysosomal gene transcription that undergoes nuclear translocation upon apilimod treatment, did not reveal any difference between *Inpp4b*^*+/+*^ and *Inpp4b*^*−/−*^ MEF (Fig. 4, *A* and *B*). Next, transcript levels of representative lysosomal genes including *LAMP1*, *MCOLN1*, *CTSB*, *CTSD*, *ATP6V1D,* and *ATP6V1H* were measured by TaqMan qPCR after vehicle or apilimod treatment. In vehicle-treated cells, *Inpp4b*^*−/−*^ MEF demonstrated modest, but significantly reduced expression of all lysosomal genes, except for *CTSD* (Fig. 4*C*). Strikingly, apilimod treatment led to a ∼3-fold increase of all lysosomal transcripts tested in *Inpp4b*^*−/−*^ MEF, whereas *Inpp4b*^*+/+*^ MEF showed no significant changes under the same conditions (Fig. 4*C*). Immunoblotting of representative lysosomal proteins demonstrated that *Inpp4b*^*−/−*^ MEF have reduced steady state expression of LAMP1, pre- and mature-cathepsin B, and V-ATPase V1H, similar to transcript expression profiles (Fig. 4, *D* and *E*). However, apilimod treatment of *Inpp4b*^*+/+*^ MEF demonstrated elevated levels of the lysosomal membrane-bound LAMP1 and V-ATPase V1H proteins, but no significant change in cathepsin proteins. By contrast*,* apilimod treatment demonstrated significantly elevated expression of all lysosomal proteins tested in *Inpp4b*^*−/−*^ MEF (Fig. 4, *D* and *E*). To quantify the differential effects of apilimod on lysosomal proteolytic function, we used the membrane permeable cathepsin B substrate-Magic Red which upon hydrolysis forms membrane impermeable fluorescent cresyl-violet and accumulates within functional lysosomes (37). The results of this assay paralleled the transcript and protein expression levels of cathepsin B, where apilimod treatment had no effect in *Inpp4b*^*+/+*^ MEF, and in *Inpp4b*^*−/−*^ MEF, despite having a reduced steady state cathepsin B activity, apilimod treatment led to a more than 3-fold induction of cathepsin B activity (Fig. 4, *F* and *G*). Although activation of nuclear translocation of TFEB by apilimod was no different in *Inpp4b*^*+/+*^ and *Inpp4b*^*−/−*^ MEF, lysosomal gene expression, protein expression, and proteolytic function of lysosomes are differentially impacted by apilimod treatment in *Inpp4b*^*+/+*^ and *Inpp4b*^*−/−*^ MEF.

### Inhibition of autophagy by apilimod is potentiated in Inpp4b-deficient cells

**Figure 5:**
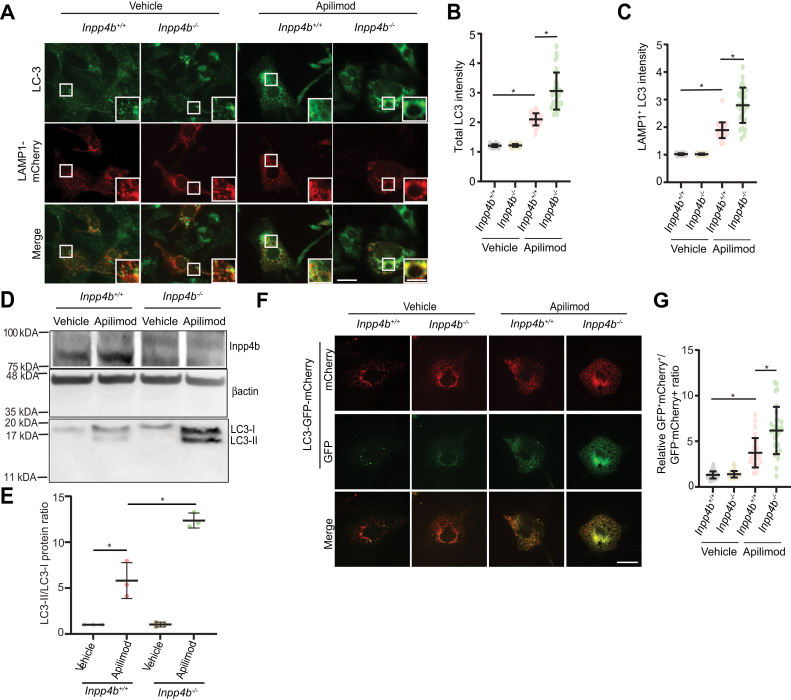
**Autophagic flux is blocked upon PIKfyve inhibition in *Inpp4b*-deficient cells.***A*, *Inpp4b*^*+/+*^ or *Inpp4b*^*−/−*^ MEFs transiently expressing LAMP1-mCherry to mark lysosomes and treated with vehicle or apilimod 10 nM for 48 h, followed by immunostain against LC3. *B*, quantifications of LC3 puncta intensity per cell across indicated conditions to measure autophagosome levels and (*C*) LC3 intensity overlapping on LAMP1-positive lysosomes where increased LC-3/LAMP1 intensity ratio indicate autolysosome formation. *D*, immunoblot of *Inpp4b*^*+/+*^ or *Inpp4b*^*−/−*^ MEFs treated with vehicle or apilimod 10 nM for 48 h and assessed for protein levels of Inpp4b, LC3, and Beta actin as loading control. *E*, quantification of immunoblot from (*D*) for LC3-II/LC3-I protein ratio shown as mean ± SD from three independent experiments. *F*, *Inpp4b*^*+/+*^ or *Inpp4b*^*−/−*^ MEF transiently expressing *mCherry-EGFP-LC3B* and treated with vehicle or apilimod 10 nM for 48 h. *G*, quantification of LC-3 green puncta over red puncta intensity ratio. The scale bar represents 15 μm, zoomed inset: 5 μm. Data represent mean ± SD from three independent experiments, with 25 to 30 cells assessed per treatment condition per experiment for (*A*–*C*) and (*F*–*G*). Statistical significance was measured by ANOVA and multiple Student’s *t* test and represented as ∗ (*p* < 0.05). PIKfyve, Phosphoinositide Kinase, FYVE-Type Zinc Finger Containing; INPP4B, inositol polyphosphate 4-phosphatase type II; MEF, mouse embryonic fibroblast.

PIKfyve inhibition has been demonstrated to inhibit autophagy in various physiological and pathological settings upon extended PIKfyve inhibition, but not upon acute exposure (12, 16, 38). We evaluated how apilimod treatment affected autophagy in the context of *Inpp4b* deficiency by first measuring levels of MAP1LC3A/B (LC3), a membrane protein specifically expressed on autophagosomes by IF with anti-LC3 antibodies (39). Total cellular LC3 staining was similar in vehicle-treated MEF. Apilimod treatment induced LC3 levels to significantly higher levels in *Inpp4b*^*−/−*^ MEF than in *Inpp4b*^*+/+*^ MEF (Fig. 5, *A* and *B*). Notably, the corresponding elevation in autophagosome levels induced by apilimod in *Inpp4b*^*−/−*^ MEF was fully rescued by ectopic INPP4B expression, indicating that LC3 induction levels are restricted by Inpp4b (Fig. S6, *A*–*C*). We also assessed extent of autolysosome formation by quantitating colocalization LAMP1-mCherry fluorescence and LC-3 by IF. Relative to vehicle-treated MEF, colocalization of LAMP1 (lysosomes) and LC3 (autophagosomes) was elevated upon apilimod treatment, and *Inpp4b*^*−/−*^ MEF had significantly higher levels of colocalization than *Inpp4b*^*+/+*^ MEF (Fig. 5, *A* and *C*). Autophagy was also assessed by measuring LC-3 status by immunoblotting (40). During autophagy, cytosolic LC3-I (∼16 kDa) is conjugated with phosphatidylethanolamine on the phagophore membrane to form LC3-II (∼14 kDa). LC3-II is absent in vehicle-treated *Inpp4b*^*+/+*^ and *Inpp4b*^*−/−*^ MEF (Figs. 5, *D* and *E* and S7), however apilimod treatment significantly induced LC3-II levels in *Inpp4b*^*+/+*^ MEF, and even further induction was observed in *Inpp4b*^*−/−*^ MEF (Figs. 5, *D* and *E* and S7). Similar observations were made in U2OS cells where INPP4B expression was silenced with siRNA (Fig. S2*E*).

Autophagy was further assessed by estimating autophagic flux using a reporter system which expresses a fusion of LC3 protein with the acid-insensitive mCherry protein and the acid-sensitive GFP protein (41, 42). This reporter permits the estimation of autophagic flux—autophagosome to autolysosome transition—by measuring the ratio of yellow (mCherry^+^ GFP^+^) or red (mCherry^+^ GFP^-^) puncta, respectively (12, 43, 44). In vehicle-treated *Inpp4b*^*+/+*^ and *Inpp4b*^*−/−*^ MEF, we observed predominantly red fluorescence indicating functional transition through autophagy (Fig. 5, *F* and *G*). By contrast, apilimod treatment resulted in predominantly yellow puncta in *Inpp4b*^*+/+*^ MEF and an even higher ratio of yellow to red puncta in *Inpp4b*^*−/−*^ MEF. (Fig. 5, *F* and *G*). Notably, the accumulation of yellow fluorescence (mCherry^+^ GFP^+^) in apilimod-treated *Inpp4b*^*−/−*^ MEF appear to be on the membranes of enlarged vacuoles. These data demonstrate that *Inpp4b* deficiency alone does not alter autophagic flux, however, apilimod treatment reveals that Inpp4b regulates autophagic flux in some conditions, including PIKfyve inhibition.

### Inpp4b deficiency exacerbates apilimod-mediated PtdIns(3)P generation

**Figure 6:**
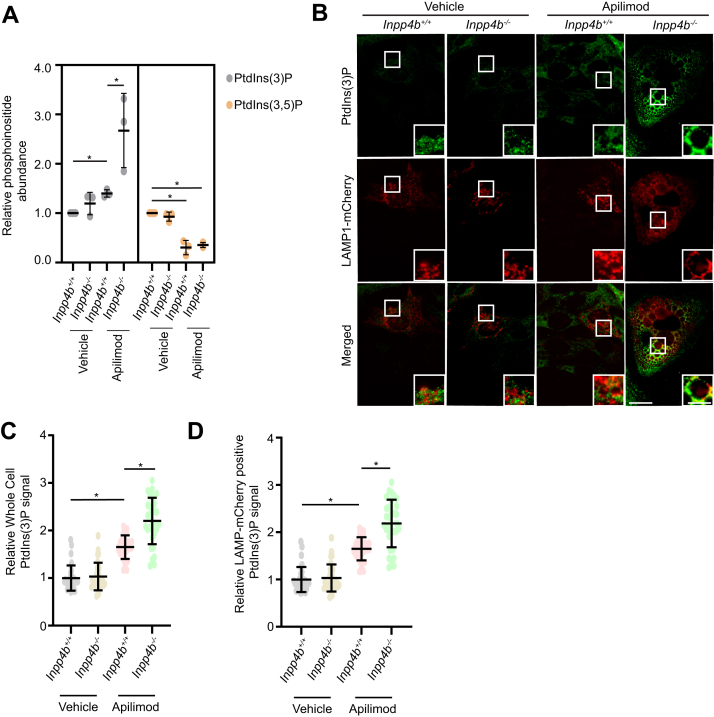
**PIKfyve inhibition in *Inpp4b*-deficient cells leads to elevated PtdIns(3)P levels**. *A*, ^3^H-*myo*-inositol incorporation and HPLC scintillation quantification of PtdIns(3)P and PtdIns(3,5)P_2_ for *Inpp4b*^*+/+*^ or *Inpp4b*^*−/−*^ MEFs treated with vehicle or apilimod (n = 3). *B*, *Inpp4b*^*+/+*^ or *Inpp4b*^*−/−*^ MEFs transiently expressing LAMP1-mCherry and treated with vehicle or apilimod 10 nM for 48 h. *C*, total cell PtdIns(3)P or (*D*) LAMP1-mCherry positive PtdIns(3)P within a cell. The scale bar represents 20 μm, zoomed inset: 5 μm. Data represent ± SD from three independent experiments with 25 to 30 cells assessed per treatment condition per experiment for (*B*–*D*). Statistical significance was measured by ANOVA and multiple Student’s *t* test and represented as ∗ (*p* < 0.05). INPP4B, inositol polyphosphate 4-phosphatase type II; MEF, mouse embryonic fibroblast; PtdIns, phosphoinositide; PtdIns(3,5)P2, phosphatidylinositol-3,5-bisphosphate; PtdIns(3)P, phosphatidylinositol-3-monophosphate; PIKfyve, Phosphoinositide Kinase, FYVE-Type Zinc Finger Containing.

Given the direct roles for PIKfyve and Inpp4b on PtdIns metabolism, we assessed how PtdIns homeostasis may be differentially affected in *Inpp4b*^*+/+*^ and *Inpp4b*^*−/−*^ MEF in response to apilimod. We first measured total cell levels of PtdIns(3)P and PtdIns(3,5)P _2_ through ^3^H-*myo*-inositol labeling and HPLC scintillation and quantification (40). *Inpp4b* deficiency did not alter PtdIns(3,5)P_2_ levels, but as expected, apilimod treatment significantly reduced PtdIns(3,5)P_2_ levels in both *Inpp4b*^*+/+*^ and *Inpp4b*^*−/−*^ MEF (Fig. 6*A*).

Total steady state PtdIns(3)P levels were also similar in *Inpp4b*^*−/−*^ MEF compared to *Inpp4b*^*+/+*^ MEF; this was unexpected given that PtdIns(3)P is the product of Inpp4b catalysis. Furthermore, apilimod treatment induced a significant ∼1.3 fold increase of PtdIns(3)P in *Inpp4b*^*+/+*^ MEF and a ∼3 fold increase of PtdIns(3)P in *Inpp4b*^*−/−*^ MEFs (Fig. 6*A*). To support these surprising findings, we also measured PtdIns(3)P by IF with an anti-PtdIns(3)P antibody in cells where lysosomes were simultaneously marked with LAMP1-mCherry (Fig. 6*B*). Firstly, IF confirmed the elevated levels of total cellular PtdIns(3)P observed in apilimod-treated *Inpp4b*^*+/+*^ and *Inpp4b*^*−/−*^ MEF (Fig. 6*C*). Colocalization of PtdIns(3)P with LAMP1-mCherry revealed a similar pattern of PtdIns(3)P levels on lysosomal membranes (Fig. 6*D*). IF of PtdIns(3,4)P_2_ revealed greater total and lysosomal levels of PtdIns(3,4)P_2_, as expected in *Inpp4b*-deficient MEF (Fig. S8, *A*–*C*). Apilimod treatment had no effect on total cell or LAMP1-mCherry positive PtdIns(3,4)P_2_ levels in *Inpp4b*^*+/+*^ MEF, however, there was a small but significant reduction in PtdIns(3,4)P_2_ levels compared to steady state levels for *Inpp4b*^*−/−*^ MEF (Fig. S8, *A*–*C*). In sum, apilimod treatment led to a significant increase in total and lysosomal PtdIns(3)P in *Inpp4b*^*−/−*^ MEF, indicating an unexpected role for Inpp4b in suppressing cellular levels of PtdIns(3)P.

### Apilimod-mediated lysosome enlargement is driven by VPS34-mediated PtdIns(3)P production

**Figure 7:**
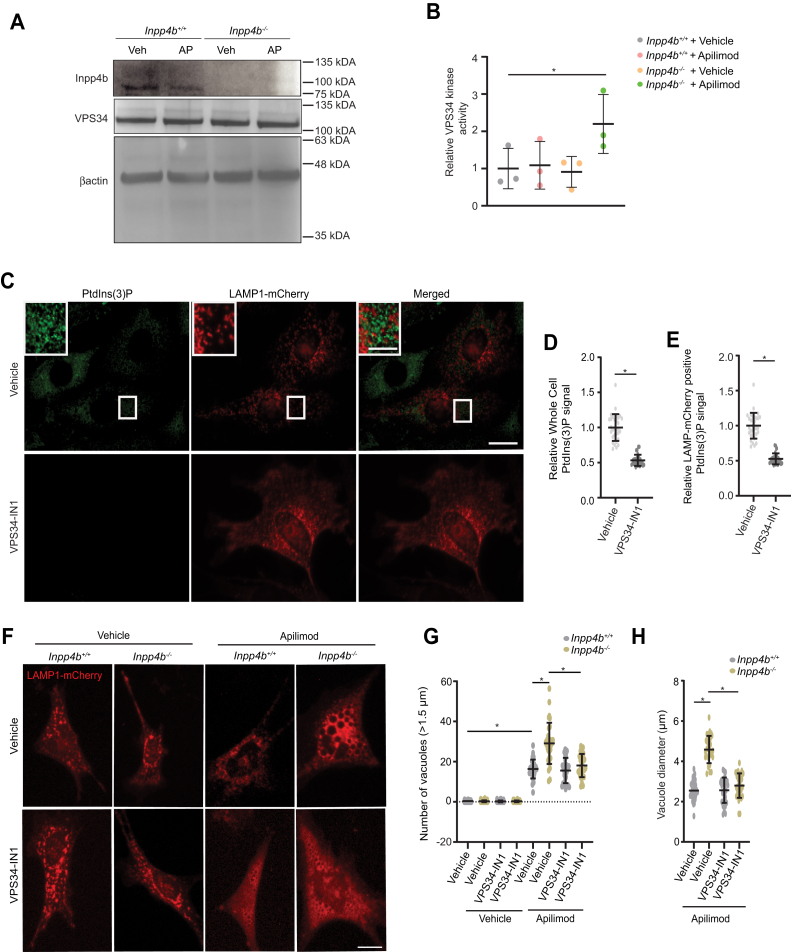
**VPS34 activity is elevated upon PIKfyve inhibition in *Inpp4b*-deficient cells.***A*, *Inpp4b*^*+/+*^ or *Inpp4b*^*−/−*^ MEFs treated with vehicle or apilimod 10 nM for 48 h, followed by immunoblotting against Inpp4b, VPS34, or Beta Actin. *B*, *Inpp4b*^*+/+*^ or *Inpp4b*^*−/−*^ MEFs treated with vehicle or apilimod 10 nM for 48 h, followed by VPS34 immunoprecipitation and kinase assay to monitor VPS34 activity. *C*, *Inpp4b*^*+/+*^ MEF transiently expressing LAMP1-mCherry and treated with vehicle or 500 nM VPS34-IN1 for 48 h followed by PtdIns(3)P immunostain. *D*, quantification from (*C*) of total cell PtdIns(3)P fluorescence signal or (*E*) PtdIns(3)P fluorescence signal overlayed on LAMP1-mCherry positive regions within a cell. The scale bar represents 20 μm, zoomed inset: 5 μm. *F*, *Inpp4b*^*+/+*^ or *Inpp4b*^*−/−*^ MEF transiently expressing LAMP1-mCherry and treated with vehicle or apilimod 10 nM or VPS34-IN1 500 nM at various combinations for 48 h. *G*, quantification of LAMP1-positive vacuoles greater than 1.5 μm in diameter per cell and (*H*) mean vacuole diameter (μm). The scale bar represents 20 μm. Data represent ± SD from three independent experiments with 25 to 30 cells assessed per treatment condition per experiment. Statistical significance was measured by ANOVA and multiple Student’s *t* test and represented as ∗ (*p* < 0.05). INPP4B, inositol polyphosphate 4-phosphatase type II; MEF, mouse embryonic fibroblast; PtdIns, phosphoinositide; PtdIns(3)P, phosphatidylinositol-3-monophosphate; PIKfyve, Phosphoinositide Kinase, FYVE-Type Zinc Finger Containing.

We sought to gain a further understanding of the mechanisms leading to elevated cellular PtdIns(3)P and its role in the exacerbated lysosomal enlargement upon apilimod treatment in *Inpp4b*^*−/−*^ MEF. Given its major role in cellular PtdIns(3)P generation, we explored VPS34 functions (45). No changes in VPS34 protein levels were observed by immunoblot in *Inpp4b* deficiency nor in vehicle- or apilimod-treated conditions (Fig. 7*A*). Surprisingly, measurement of the lipid kinase activity of VPS34 demonstrated a significant elevation only in apilimod-treated *Inpp4b*^*−/−*^ MEF (Fig. 7*B*), thereby explaining elevated PtdIns(3)P levels.

To elucidate a role for PtdIns(3)P in the formation of massively enlarged vacuoles in apilimod-treated *Inpp4b*^*−/−*^ MEF, we reasoned that depletion of cellular PtdIns(3)P could attenuate this phenotype. Thus, we treated MEF with both apilimod and the specific VPS34 inhibitor, VPS34-IN1 (25, 46). PtdIns(3)P IF performed on VPS34-IN1-treated MEF demonstrated that VPS34 inhibition can effectively diminish total cellular and lysosomal localized PtdIns(3)P levels (Fig. 7, *C*–*E*). Notably, VPS34 inhibition in apilimod-treated *Inpp4b*^*−/−*^ MEF rescued the exacerbated lysosome enlargement but only to levels of enlargement normally observed in *Inpp4b*^*+/+*^ MEF; VPS34-IN1 had little effect on the enlarged lysosome size and number in apilimod-treated *Inpp4b*^*+/+*^ MEF, indicating that induction of PtdIns(3)P levels may not be necessary or sufficient for normal apilimod responses (Fig. 7, *F*–*H*). In sum, VPS34-IN1 revealed that aberrant hyperactivation of VPS34 in *Inpp4b*^*−/−*^ MEF treated with apilimod leads to exacerbated lysosomal enlargement. These findings suggest the existence of a role for Inpp4b in the regulation of VPS34 activation, and thereby PtdIns(3)P levels, in cells undergoing lysosomal stress induced by PIKfyve inhibition.

## Discussion

Lysosomes, the primary catabolic organelles in the cell, play pivotal roles in many cellular processes including cell differentiation, plasma membrane repair, programmed cell death, nutrient sensing, and stress responses (47). Thus, exquisite control of lysosome homeostasis, including the dynamic mechanisms that control total lysosomal content, number, and size are critical to maintaining normal lysosomal and cellular functions. Emerging data indicates key roles for PtdIns in maintaining lysosomal homeostasis. In this study, we explored the dynamics of PIKfyve inhibition and resultant PtdIns(3,5)P_2_ depletion, which leads to disruption of lysosomal “fusion-fission” cycles by compromising “fission”, thereby generating enlarged coalescent lysosomes (11, 16, 17). PIKfyve inhibition also disrupts endocytic cargo delivery, autophagosome formation, and autophagic flux, possibly a result of lysosome membrane perturbations where lysosomes fail to resolve from other lysosomes, late endosomes, and autophagosomes (11, 12). PIKfyve has been shown to functionally coordinate with the Class III PI3-Kinase VPS34 through its catalytic product PtdIns(3)P, which serves as a membrane localization target for PIKfyve, as well as a substrate precursor for PtdIns(3,5)P_2_ synthesis (45). Together, the functions of PIKfyve and VPS34 recruit and regulate components of endosomal and lysosomal recycling (48). This PIKfyve-VPS34 crosstalk suggests the existence of other PIKfyve cross talks with other PtdIns-modifying enzymes.

Emerging studies present a role for the lipid-phosphatase INPP4B in the regulation of lysosome-associated functions. Our group has demonstrated that *INPP4B* expression in acute myeloid leukemia regulates lysosomal biogenesis and functions which are crucial for leukemia stem cell maintenance, differentiation, and chemoresistance (29). Another study demonstrated that INPP4B depletion in triple negative breast cancer resulted in delayed EGFR trafficking from early endosomes to late endosomes/lysosomes (49). A role for INPP4B in lysosomal functions is further supported by the observation that elevated expression of INPP4B in *PIK3CA*-mutant ER^+^ breast cancer cells induce formation of late endosomes and lysosomes, increase cargo trafficking toward lysosomes, and promote endosomal sequestration and lysosomal degradation of key signaling proteins (28). Together, these data support a role for INPP4B in the enhancement of function and cellular content of lysosomes. Indeed, this study further supports this notion as *Inpp4b*-deficient MEF consistently demonstrated reduced total lysosomal content and lysosome numbers as measured by LAMP1 IF, lysotracker labeling, and lysosomal accumulation of LY (Figs. 1, 2 and S1). Furthermore, lysosomal transcript and protein expression levels were reduced in *Inpp4b*^*−/−*^ MEF, together suggesting a role for Inpp4b in promoting and/or maintaining lysosomal content by controlling biogenesis through transcriptional mechanisms (Fig. 4). Since INPP4B and PIKfyve both regulate lysosome function, we sought to shed light on putative interactions between these two enzymes.

Apilimod-mediated PIKfyve inhibition in MEF leads to formation of numerous enlarged translucent cytoplasmic vacuoles readily seen by light microscopy. In previous studies, 20 nM apilimod for 1 h in RAW macrophages led to rapid lysosome enlargement to a volume of ∼10 μm^3^. HeLa or RPE cells required 100 to 200 nM apilimod to generate lysosome of 3 to 4 um^3^ (16, 17). By comparison, apilimod treatment of *Inpp4b*^*+/+*^ MEF at 10 nM or 500 nM for 1 h dilated lysosome volume to 3 um^3^ or 4.5 um^3^, respectively. The key findings made in this study stem from the observations that apilimod treatment of *Inpp4b*^*−/−*^ MEF generate significantly larger cytoplasmic vacuoles than apilimod-treated *Inpp4b*^*+/+*^. This observation was the first indication of a putative crosstalk between INPP4B and PIKfyve. To gain further insight into the exacerbated vacuolation phenotype in *Inpp4b-deficient* MEF, we first confirmed that enlarged vacuoles were of lysosomal origin as proposed by Choy et al. (17). Loading of MEF with LY prior to apilimod stimulation permitted effective lysosomal accumulation, which then enabled the clear visualization and quantitation of lysosomal features after apilimod treatment. LY labeling confirmed that lysosome size and number were affected to a greater degree in *Inpp4b*^*−/−*^ MEF by apilimod; and that apilimod inhibits lysosomal accumulation of cargo, a phenotype which was also exacerbated in *Inpp4b*^*−/−*^ MEF. Lysosome dynamics in *Inpp4b*^*−/−*^ MEF were exquisitely sensitized to apilimod, as demonstrated by the severely compromised lysosomal fission, relative to the moderately compromised fission rates observed in *Inpp4b*^*+/+*^ MEF.

In our attempts to further understand the underlying biology responsible for the exaggerated response to apilimod observed in *Inpp4b*^*−/−*^ MEF, we observed that the expression of representative lysosomal gene transcripts were either unchanged or decreased in *Inpp4b*^*+/+*^ MEF treated with apilimod. *Inpp4b*^*−/−*^ MEF on the other hand demonstrated a significant 2-3-fold increase in the expression of all lysosomal transcripts tested. Elevated lysosomal biogenesis observed in *Inpp4b*^*−/−*^ MEF was not explained by the nuclear localization of Tfeb; however, altered Tfeb transactivation activity by phosphorylation and acetylation by the action of factors including mTORC1, PKC, PKD, GSK3β, and phosphatases such as the Ca2+-dependent calcineurin cannot be formally excluded with our data (50, 51, 52). Analysis of lysosomal protein levels showed that lysosomal membrane–embedded proteins including Lamp1 and V-ATPase (V1H) subunit were significantly elevated in *Inpp4b*^*+/+*^ and *Inpp4b*^*−/−*^ MEF upon apilimod treatment. On the other hand, cathepsin-B protein levels and activity are elevated only in apilimod-treated *Inpp4b*^*−/−*^ MEF, suggesting that the stability of some lysosomal proteins may also be differentially regulated in apilimod-treated *Inpp4b*^*+/+*^ and *Inpp4b*^*−/−*^ MEF. Disruption of autophagic flux, which blocks turnover of lysosomal proteins (12, 38) may explain elevation of some lysosomal proteins (LAMP1 and V-ATPase). Also, it is currently unclear if elevated cathepsin-B protein and activity result due to disrupted feedback regulation from apilimod effects. In sum, the specific consequences of Inpp4b deficiency combined with PIKfyve inhibition on gene expression require further study.

Our data also show that apilimod can elevate autophagosome levels and promote colocalization with lysosomes to form autolysosomes, however, autolysosome maturation appears to be inhibited as indicated by reduced autophagic flux. Interestingly, enlarged lysosomes in apilimod-treated cells are proteolytically competent as measured by Magic Red and DQ-BSA suggesting that altered lysosomal membrane composition may confer resistance to membrane degradation or other membrane-associated processes required for autolysosome maturation. Our observations are consistent with previous studies regarding the consequences of apilimod (12, 53, 54, 55), with the exception that *Inpp4b*^*−/−*^ MEF display exacerbated versions of this phenotype.

**Figure 8:**
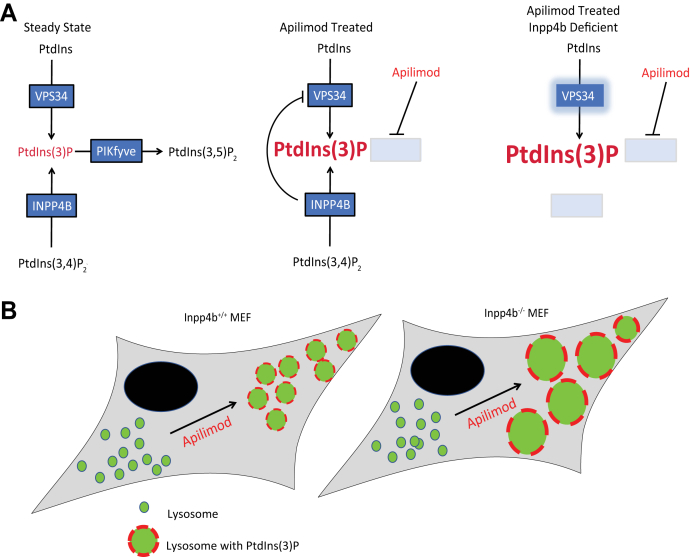
**Model for lysosome enlargement upon PIKfyve inhibition in *Inpp4b* deficient cells.***A*, in the steady state, cycling of phosphoinositides involve VPS34-dependent conversion of phosphatidylinositol (PtdIns) to PtdIns(3)P, PtdIns(3,4)P_2_ conversion by Inpp4b to PtdIns(3)P, and PtdIns(3)P conversion by PIKfyve to PtdIns(3,5)P_2_. Apilimod-induced PIKfyve inhibition deplete PtdIns(3,5)P_2_ to elevate the substrate PtdIns(3)P, and simultaneous PIKfyve and Inpp4b suppression further augment PtdIns(3)P levels. *B*, PIKfyve inhibition in *Inpp4b*^*+/+*^ MEF induce moderate lysosome enlargement, where lysosomes coalesce to become fewer in number and greater in individual lysosome volume. PIKfyve inhibition in *Inpp4b*^*−/−*^ MEF exacerbate lysosome enlargement, where lysosomes are much greater in individual volume and this enlargement is facilitated by enrichment of VPS34-induced membrane PtdIns(3)P. INPP4B, inositol polyphosphate 4-phosphatase type II; MEF, mouse embryonic fibroblast; PtdIns, phosphoinositide; PtdIns(3,5)P2, phosphatidylinositol-3,5-bisphosphate; PtdIns(3)P, phosphatidylinositol-3-monophosphate; PIKfyve, Phosphoinositide Kinase, FYVE-Type Zinc Finger Containing.

Finally, we measured intracellular PtdIns levels in apilimod-treated MEF. PtdIns(3)P levels were observed to be unchanged in vehicle-treated MEF, which suggests that other major mechanisms govern PtdIns(3)P levels, and Inpp4b may only be a minor contributor. As previously reported, apilimod leads to moderately elevated levels of PtdIns(3)P, which we also observed in *Inpp4b*^*+/+*^ MEF (56). Surprisingly, apilimod treatment in *Inpp4b*^*−/−*^ MEF led to a dramatic increase in both total cellular and lysosomal levels of PtdIns(3)P. This observation was deemed paradoxical given that *Inpp4b* deficiency should generate less PtdIns(3)P and suggests that Inpp4b may regulate PtdIns(3)P levels through an indirect mechanism. To explain elevated PtdIns(3)P, our tests revealed that VPS34 activity, but not protein levels, was elevated. Furthermore, VPS34-IN1, a specific inhibitor of VPS34, depleted PtdIns(3)P and reversed the exacerbated lysosomal enlargement observed with *Inpp4b* deficiency. These data point to a direct role for VPS34 in apilimod-mediated PtdIns(3)P induction (Fig. 8). Furthermore, our results highlight a novel regulatory signaling axis linking PIKfyve, VPS34, and Inpp4b functions.

In conclusion, our study demonstrates that *Inpp4b* deficiency sensitizes cells and lysosomes to the plethora of effects conferred by apilimod-mediated PIKfyve inhibition, the most obvious of which is significantly exacerbated lysosomal enlargement compared to *WT* cells, most likely due to blocked fission and thus, enhanced lysosome coalescence. Similar exacerbated consequences are observed for various other phenotypes associated with PIKfyve inhibition including disrupted autophagy and reduced cargo delivery to lysosomes (11, 12, 13, 14). Finally, we have uncovered a novel role for Inpp4b in regulating the activation of VPS34 activity and induction of PtdIns(3)P levels, which was only revealed upon lysosomal stress activated by PIKfyve inhibition. This function is mediated through a novel PIKfyve–VPS34–Inpp4b regulatory signaling axis.

## Experimental procedures

### MEF preparation, cell culture conditions, transfections, drug treatment

Immortalized *Inpp4b*^*+/+*^ and *Inpp4b*^*−/−*^ MEF were generated as previously detailed (51). MEF and U2OS cells were maintained in Dulbecco’s Modified Eagle’s Medium (DMEM) supplemented with 10% Fetal Bovine Serum (FBS). MEF or U2OS cells were transiently transfected with *pTWIST-mCherry, pTWIST-Inpp4b-mCherry, mCherry-Lamp1, mCherry-EGFP-LC3B, pEGFP, GFP-Inpp4b, GFP-Inpp4b (C845A),* and *pEGFP-TFEB*. U2OS cells were stably transfected with *mCherry-Lamp1* through selection with 200 μg/ml G418 for 10 days. Transfections of MEF and U2OS cells performed with Fugene HD (Promega) at 3:1 of DNA:Fugene ratio for 24 h followed by washing and supplementation with complete DMEM growth media. siRNA-mediated gene silencing for *INPP4B* in U2OS cells carried out using DharmaFECT1 Transfection reagent (GE Dharmacon). Briefly, 0.1 nmol of nontargeting or *Inpp4b* siRNA (GE Dharmacon) mixed with 2 μl of DharmaFECT1 Transfection reagent in DMEM media without FBS was added to U2OS cells for 24 h, followed by washing off the transfection mix with PBS and growth of cells for 48 h with treatment before imaging and Western blot. MEF and U2OS cells treated with apilimod or VPS34-IN1 (Selleck Chemicals) to inhibit PIKfyve or VPS34 functions respectively at doses and durations indicated.

### Retroviral transduction

3.0 × 10^6^ HEK 293T cells were grown in a 10 cm dish for 24 followed by calcium phosphate transfection. Briefly, 10 μg of retroviral plasmid *pWZL hygro SV40 T-Large* was mixed with 5 μg of pCL-Eco retroviral packaging vector and 2M CaCl_2_ to a final volume of 300 μl in sterile water. The transfection mix was supplemented with equal volume of 2× Hepes-buffered saline (140 mM NaCl, 1.5 mM Na_2_HPO_4_) followed by addition to the cells. Following 24 h post transfection, the media was changed and supplemented with complete DMEM growth media. Media was collected 48 h and 72 h post transfection. Virus-enriched media was filtered through a 0.45-micron filter, supplemented with 8 μg/ml protamine sulfate, and added to MEF cells grown in 10 cm dishes. Infections were repeated every 8 h and MEF cells were selected for 4 days with 75 μg/ml hygromycin B.

### Lysosome labeling

MEF cell lysosomes were labeled with 1 mg/ml LY (Thermo Fisher Scientific) for 2 h in complete growth media at 37 °C and 5% CO_2_, followed by washing in PBS and supplementation of complete media for 1 h. LTR (Thermo Fisher) was also used to label MEF cell lysosomes by incubation at 1 μM for 30 min in complete growth media. Magic Red (Abcam) was used to assess lysosomal cathepsin B activity in MEF cells by incubation for 1 h according to manufacturer instructions.

### Immunofluorescence

Immunolabeling of cells following apilimod treatment was performed by fixation with 4% (v/v) paraformaldehyde for 15 min, permeabilization with 100% ice-cold methanol for 5 min, and blocking in 3% bovine serum albumin (v/v) in PBS. Cells were incubated with rabbit monoclonal antibody against mouse LC3B (1:200; Cell Signaling) and Alexa Fluor 488-conjugated goat polyclonal antibody against rabbit IgG (1:1000; Thermo Fisher). Alternatively, immunostaining was performed with rat monoclonal antibody against mouse LAMP1 (1:200, Clone 1D4B; Thermo Fisher) and Dylight 488-conjugated donkey polyclonal antibody against rat IgG (1:1000; Bethyl, Montgomery, TX). For CD63 immunostaining, fixations were performed with 4% (v/v) paraformaldehyde for 15 min, followed by permeabilization with 0.1% Triton X-100 for 10 min at room temperature. Immunostaining was performed with mouse monoclonal antibody against mouse CD63 (1:150; Novus Biologicals) and Dylight 488-conjugated Goat polyclonal antibody against mouse IgG (1:1000; Bethyl). Total cell PtdIns(3,4)P_2_ or PtdIns(3)P immunostaining was performed by fixation with 4% (v/v) paraformaldehyde for 15 min, permeabilization with 20 μM digitonin (Promega) in buffer A (20 mM Pipes pH 6.8, 137 mM NaCl, 2.7 mM KCl) for 30 min, and blocking with buffer A containing 5% normal goat serum and 50 mM NH_4_Cl. Immunostaining was performed with anti-PtdIns(3,4)P_2_ IgG or anti-PtdIns(3)P IgG (Echelon) and Dylight 488-conjugated Goat polyclonal antibody against mouse IgG (1:1000; Bethyl). Samples are mounted onto microscope slides through DAKO fluorescent mounting media and imaged.

### Live- and fixed-cell microscopy

Manual assessment of vacuole and lysosome size, number, LAMP1 positive vesicles, TFEB-GFP localization, Magic Red, or LTR-stained lysosomes were performed using EVOS-FL fluorescent inverted microscope controlled by EVOS XL core imaging system at 20× 0.4 NA. (Thermo Fisher) objective. Spinning disc confocal microscopy was used to perform live imaging through Olympus IX81 inverted microscope connected to Hamamatsu C9100-13 EMCCD camera with 60× 1.35 NA. objective and controlled by Volocity 6.3.0 (PerkinElmer). Time lapse live imaging performed with an environmental chamber set to 37 °C and 5% CO_2_ in DMEM complete media. Fixed cells were observed through ZEISS AxioImager M2 Epifluorescence microscope connected to AxioCam MRm CCD camera and controlled by AxioVision Software Version 4.8 at 20× 0.8 NA. or 40× 1.4 NA. objective (Carl Zeiss).

### Image analysis

To identify vacuoles as LAMP-1+ and of lysosomal origin, first, we set a minimum size threshold exclusion parameter to identify vacuoles as being greater than 1.5 μm in diameter on light microscopy using ImageJ. LAMP-1-mCherry fluorescence was then overlaid on these enlarged vacuoles and percentage LAMP-1-mCherry positivity was calculated. To measure the percentage of cells with nuclear TFEB, cells were scored to have nuclear TFEB if the nucleus had greater intensity than cytosol using ImageJ. To measure the percentage of lysosomes filled with LY per cell, images imported into ImageJ and number of LAMP1-positive vesicles were manually scored for presence of LY signal within the lysosome lumen.

To quantify LAMP1 or LC3 immunostaining through ImageJ, intensity thresholding was applied to identify fluorescent structures and the mean intensity was obtained for each cell. To quantify LC3 intensity over LAMP1-positive structures, ImageJ was used to threshold for LAMP1-mCherry signal and generating a mask, which was applied to the green (LC3 immunostain) channel to measure LC3 intensity on LAMP1-mCherry positive regions. For MEF cells transiently expressing mCherry-eGFP-LC3B*,* similar approach was used to determine LC3 green puncta intensity over LC3 red puncta structures where relative intensity ratio greater than 1 indicate formation of autolysosomes due to reduced autophagic flux. Similar image analysis technique was applied for MEF cells transiently expressing LAMP1-mCherry to evaluate PtdIns(3,4)P_2_ or PtdIns(3)P levels overlayed on LAMP1-mCherry positive regions within a cell. To quantify CD63 puncta number per cell, intensity thresholding was similarly applied to identify fluorescent puncta structures followed by total CD63 puncta number per cell.

To analyze lysosome-to-cytosol intensity ratio of INPP4B-mCherry, images were imported into ImageJ, and lines were assigned to nucleus-excluded areas of a cell measuring 40-pixel in length and 5-pixel in width. Intensity plot profiles were acquired and exported into excel spreadsheet. Intensity arranged according to values and ratio obtained for highest 10 pixels over lowest 10 pixels (F_H_/F_L_ fluorescent ratio), where ratio values of approximately 1 represent cytosolic distribution.

To measure lysosome volume and number per cell, particle detection and volumetric tools from Volocity 6.3.0 were used. Briefly, Z-stack images imported into Volocity and punctate lysosome structures identified by applying a 2× cytosol intensity threshold to exclude cytosol and background. Further criteria to include particles greater than 0.3 μm^3^ removed noise-derived particles. Each cell was isolated by drawing region interest for individual cell analysis. Quantification of lysosome splitting frequency was performed through Imaris (BitPlane) using ‘ImarisTrackLineage’ module, where lysosome splitting was defined as frequency of events where two particles were produced from a single particle.

### VPS34 immunoprecipitation and VPS34 kinase assay

Following vehicle or apilimod treatment of *Inpp4b*^*+/+*^ and *Inpp4b*^*−/−*^ MEF cells, VPS34 immunoprecipitation was performed using the protocol from Cell Signaling (#4263 from Cell Signaling). Five hundred microgram of total protein lysate was incubated with VPS34 antibody #4263 (1:50) overnight at 4 °C. Precleared protein A magnetic beads (Cell Signaling) was incubated with immunocomplex for 20 min at room temperature on a shaking rotator. The magnetic beads were washed five times with TBST buffer using DynaMag-2 Spin Magnet (Thermo Fisher). Kinase assay performed on immunoprecipitated VPS34 using Class III PI3K ELISA Kit (K-3000, Echelon) through the protocol recommended for beads conjugated to enzyme. Kinase assay and PI3P production was performed at 30 °C for 1 h at 1100 r.p.m. Solution-containing PI3P was isolated from the magnetic beads using DynaMag-2 Spin Magnet and used for colorimetric detection of PI3P on ELISA plates.

### Generation of NeuroMab Inpp4b antibody

Hybridoma cells that secrete NeuroMab clone N171/17-anti-INPP4b antibody were cultured in DMEM (Gibco) supplemented with 10% FBS (Gibco) and 1% penicillin-streptomycin (Gibco). Cells were passaged every 2 days up until 7 days before collection of antibody containing media by centrifugation at 200*g*. The antibody-containing media was directly applied to nitrocellulose membrane for Inpp4b detection.

### Western blot

Whole cell lysates were generated using 1× RIPA buffer supplemented with protease inhibitor. Proteins were immunoblotted with the antibodies anti-LC3B (#3868), beta actin (#4967), INPP4B (#14543), LAMP1 (#3243) from Cell Signaling, anti-vATPase V1H (sc-166227), and cathepsin B (sc-365558) from Santa Cruz. Immunoblotting and immunoprecipitation of VPS34 was performed using the following antibody: Rabbit monoclonal antibody for PI3 Kinase Class III (1:1000 for Western blot or 1:50 for immunoprecipitation, 4263, Cell Signaling). Representative western blots used for figure generation are listed for visualization (Fig. S9).

### Flow cytometry

MEF cells were incubated with 10 μg/ml DQ-BSA (Invitrogen) or 1 mg/ml LY (Thermo Fisher) for 1 h to 6 h at 37 °C, or LTR 1 μM for 30 min, or Magic red for 1 h. Alternatively, LAMP1-mCherry signal of U2OS cells are recorded through flow cytometry following apilimod treatment. Briefly, cells were washed twice with PBS at each time point and whole cell fluorescence was recorded with the Beckman Coulter Cytoflex flow cytometer (Beckman). A total of 10,000 events was counted per condition per sample using the fluorescein isothiocyanate channel for DQ-BSA and LY or phycoerythrin channel for LTR and LAMP1-mCherry and Magic red. Background signal was determined from nonlabeled cells at time 0.

### PtdIns labeling with ^3^H-myo-inositol and HPLC-coupled flow scintillation

MEF cells were incubated for two 24 h cycles with inositol-free media (MP Biomedical), 10% dialyzed FBS (Gibco), 4 mM L-glutamine (Sigma Aldrich), 1× insulin-transferrin-selenium-ethanolamine (Gibco), 20 μCi/ml myo-[2-^3^-H(N)] inositol (PerkinElmer) and indicated treatment conditions. Cells were washed twice with 1× PBS between each 24 h cycle. Lipid precipitation induced, followed by lipid deacylation, extraction, and PtdIns separation by HPLC (Agilent Technologies) through anion-exchange 4.6 × 250-mm column (Phenomenex) as previously mentioned (CH *et al*., 2018). β-RAM 4 (LabLogic) and 1:2 ratio of eluate to scintillation fluid (LabLogic) was used to detect radiolabeled eluate, followed by analysis with Laura 4 software (Ho *et al*., 2016).

### Quantitative RT PCR

RNA isolation from MEF cells was performed through Qiagen RNeasy mini kit (Qiagen). Superscript IV Vilo cDNA synthesis kit (Thermo Fisher) was used to reverse transcribe equal amount of mRNA. The resulting cDNA was amplified through quantitative PCR using TaqMan Fast Advanced Master mix (Applied Biosystems) according to manufacturer instructions in presence of Taqman assays with QuantStudio 3 Real-Time PCR system (Thermo Fisher) controlled by QuantStudio Design and Analysis Software version 1.2 (Thermo Fisher). Taqman assays include Actb (Mm02619580_g1), CtsD (Mm00515586_m1), CtsB (Mm00514443_g1), Atp6v1h (Mm01224350_m1), Atp6v1d (Hs00211133_m1), Lamp1 (Mm01217068_g1), and Mcoln1 (Mm01211241_g1) and were performed in triplicates. Relative quantification (ΔΔCt method) was used to determine gene expression normalized to Actb and vehicle-treated WT MEF.

### Statistical analysis

All experiments are conducted independently at least three times. All error bars represent SD. Statistical analysis to compare significance between multiple conditions were performed by ANOVA and post hoc analyses were performed with multiple Student’s *t* test with Bonferroni correction. *p* values less than 0.05 considered were statistically significant.

## Data availability

Upon Request

## Supporting information

This article contains supporting information.

## Conflict of interests

The authors declare no competing or financial interests.

## Supporting information

Supplementary Movie 1 **Lysosome dynamics for vehicle treated *Inpp4b***^***+/+***^**MEF cells**. Live cell imaging performed for *Inpp4b*^*+/+*^ MEF cells pre-labeled with Lucifer yellow, treated with vehicle and single z-plane images acquired every 20 s for 15 min. Indicated are scale and time

Supplementary Movie 2 **Lysosome dynamics for apilimod treated *Inpp4b***^***+/+***^**MEF cells**. Live cell imaging performed for *Inpp4b*^*+/+*^ MEF cells pre-labeled with Lucifer yellow, treated with apilimod 10 nM for 1 h and single z-plane images acquired every 20 s for 15 min. Indicated are scale and time

Supplementary Movie 3 **Lysosome dynamics for vehicle treated *Inpp4b***^***−/−***^**MEF cells**. Live cell imaging performed for *Inpp4b*^*−/−*^ MEF cells pre-labeled with Lucifer yellow, treated with vehicle and single z-plane images acquired every 20 s for 15 min. Indicated are scale and time

Supplementary Movie 4 **Lysosome dynamics for apilimod treated *Inpp4b***^***−/−***^**MEF cells**. Live cell imaging performed for *Inpp4b*^*−/−*^ MEF cells pre-labeled with Lucifer yellow, treated with apilimod 10 nM for 1 h and single z-plane images acquired every 20 s for 15 min. Indicated are scale and time

Supplementary Figure S1 **Vacuole formation and rescue in Apilimod treated *Inpp4b***^***−/−***^**MEFs. (A)***Inpp4b*^*+/+*^ or *Inpp4b*^*−/−*^ MEFs transiently expressing LAMP1-mCherry to mark lysosomes followed by vehicle or 10 nM apilimod treatment for 48 h. Quantification of **(B)** Number of vacuoles (>1.5 μm in diameter) per cell across indicated treatments, and **(C)** Mean vacuole diameter (μm). Scale Bar: 20 μm, zoomed inset: 5 μm. **(D-F)***Inpp4b*^*+/+*^ or *Inpp4b*^*−/−*^ MEFs treated with vehicle or apilimod 10 nM for 48 h, followed by Lysotracker Red staining to monitor lysosome levels in Apilimod treated cells. Scale bar: 20 μm. **(G)***Inpp4b*^*−/−*^ MEFs transiently expressing *pEGFP, GFP-Inpp4b, or GFP-Inpp4b (C845A)* and treated with vehicle or apilimod 10 nM for 48 h. Transfected cells indicated by green rectangular box outline vs. non-transfected cell(s) indicated by black rectangular box outline. Scale bar: 25 μm. Quantification of **(H)** Number of vacuoles (>1.5 μm in diameter) per cell across indicated treatments, and **(I)** Mean vacuole diameter (μm). Data represent ± SD from three independent experiments with 25 to 30 cells assessed per treatment condition per experiment for A*-C* or 10,000 events per treatment condition per experiment for E. Statistical significance was measured by ANOVA and multiple Student’s *t* test and represented as ∗ (*P* < 0.05)

Supplementary Figure S2 **Effect of INPP4B and PIKfyve suppression on lysosomes and autophagosomes in U2OS cells. (A)** U2OS cells stably expressing mCherry-LAMP1 and treated with vehicle or apilimod 10 nM for 48 h in control (siCtrl) and INPP4B-silenced cells (siINPP4B). **(B)** Quantification of mCherry-LAMP1 positive vacuole number (>1.5 μm in diameter) per cell across indicated treatments, **(C)** Mean vacuole diameter (μm) positive for mCherry-LAMP1 per cell across apilimod treated conditions and **(D)** mCherry-LAMP1 intensity per cell across indicated conditions as measured through flow cytometry. Scale bar: 20 μm. **(E)** U2OS cells from *(A)* immunoblotted for INPP4B, LC-3 and beta actin. Data represent ± SD from three independent experiments with 40 to 50 cells assessed per treatment condition per experiment for *A-C* or 10,000 events per treatment condition per experiment for *D*. Statistical significance was measured by ANOVA and multiple Student’s *t* test and represented as ∗ (*P* < 0.05)

Supplementary Figure S3 **Effect of INPP4B and PIKfyve suppression on late endosomes. (A)***Inpp4b*^*+/+*^ or *Inpp4b*^*−/−*^ MEFs treated with vehicle or apilimod 10 nM for 48 followed by late endosome immunostaining with CD63 antibody. Scale bar: 20 μm. **(B)** Quantification of late endosome or CD63+ puncta number per cell across indicated conditions. Data represent ± SD from three independent experiments with 25 to 30 cells assessed per treatment condition per experiment. Statistical significance was measured by ANOVA and multiple Student’s *t* test and represented as ∗ (*P* < 0.05)

Supplementary Figure S4 **Endocytic dynamics of Lucifer Yellow in Inpp4b and PIKfyve suppressed cells. (A)***Inpp4b*^*+/+*^ or *Inpp4b*^*−/−*^ MEFs treated with vehicle or apilimod 10 nM for 48 followed by pulsing with Lucifer Yellow for 0, 1, 2 and 4 h. Quantification of Lucifer Yellow intensity as measured through flow cytometry as 10,000 events per treatment condition per experiment**. (B-D)** Representative flow cytometry plot diagrams of *Inpp4b*^*+/+*^ or *Inpp4b*^*−/−*^ MEFs from **(A)** pulsed with Lucifer Yellow for 1 h or 2 h or 4 h respectively. Statistical significance was measured by ANOVA and multiple Student’s *t* test and represented as ∗ (*P* < 0.05)

Supplementary Figure S5 **Intracellular tracking of endocytic dynamics in Inpp4b and PIKfyve suppressed cells.** (A) *Inpp4b*^*+/+*^ or *Inpp4b*^*−/−*^ MEFs transiently expressing mCherry-LAMP1 and treated with vehicle or apilimod 10 nM for 48 h. Lucifer yellow pulsed for 0, 1, 2 and 4 h. **(B)** Quantification of percentage of mCherry-LAMP1 positive vesicles filled with Lucifer Yellow. Scale bar: 25 μm. **(C)***Inpp4b*^*+/+*^ or *Inpp4b*^*−/−*^ MEFs treated with vehicle or apilimod 10 nM for 48 followed by pulsing with DQ-BSA for 0, 1, 2 and 4 h. Quantification of DQ-BSA intensity as measured through flow cytometry. Data represent + SD from three independent experiments with 25 to 30 cells assessed per treatment condition per experiment for **A-B** or 10,000 events per treatment condition per experiment for **C**. Statistical significance was measured by ANOVA and multiple Student’s *t* test and represented as ∗ (*P* < 0.05)

Supplementary Figure S6 **INPP4B expression rescue autophagosome defect from PIKfyve suppression. (A)***Inpp4b*^*+/+*^ or *Inpp4b*^*−/−*^ MEFs transiently expressing mCherry or INPP4B-mCherry and treated with vehicle or apilimod 10 nM for 48 h. **(B)** Inset represents a single cell from **(A)** expanded field of view. **(C)** Comparison between non-transfected and transfected cells for LC3 puncta intensity per cell across indicated conditions. Scale bar: 10 μm, zoomed inset: 5 μm. Data represent ± SD from three independent experiments with 25 to 30 cells assessed per treatment condition per experiment. Statistical significance was measured by ANOVA and multiple Student’s *t* test and represented as ∗ (*P* < 0.05)

Supplementary Figure S7 **Total LC3-I and LC3-II protein levels from Fig. 5D.***Inpp4b*^*+/+*^ or *Inpp4b*^*−/−*^ MEFs treated with vehicle or apilimod 10 nM for 48 h, followed by immunoblot and measurement of total **(A)** LC3-I or **(B)** LC3-II normalized against Beta-actin, or **(C)** LC3-I and LC3-II protein levels in a single graph to show relative increase of each protein under indicated treatment conditions. Statistical significance was measured by ANOVA and multiple Student’s *t* test and represented as ∗ (*P* < 0.05)

Supplementary Figure S8 **PtdIns(3,4)P**_**2**_**phosphoinositide measurements in Inpp4b and PIKfyve suppressed cells. (A)***Inpp4b*^*+/+*^ or *Inpp4b*^*−/−*^ MEFs transiently expressing LAMP1-mCherry and treated with vehicle or apilimod 10 nM for 48 h. Quantification of **(B)** Total cell PtdIns(3,4)P_2_ or **(C)** PtdIns(3,4)P _2_ fluorescence signal overlayed on LAMP1-mCherry positive regions within a cell. Scale bar: 20 μm, zoomed inset: 5 μm. Data represent ± SD from three independent experiments with 25 to 30 cells assessed per treatment condition per experiment for **A-C***.* Significance measured through multiple Student’s *t* test represented as ∗ in comparison to indicated conditions (p<0.05)

Supplementary Figure S9 **Original uncropped western blot images displayed in the manuscript.** Complete western blot images with representative ladders and molecular weight markers used to generate manuscript figures. Western blot bands outlined by black box were used for figure generation
