# Supplementary figures and images for: Inhibition of lipid kinase PIKfyve reveals a role for phosphatase Inpp4b in the regulation of PI(3)P-mediated lysosome dynamics through VPS34 activity
Source: J Biol Chem. 2022 Jun 26;298(8):102187. doi: 10.1016/j.jbc.2022.102187 (PMC9304791; doi:10.1016/j.jbc.2022.102187)

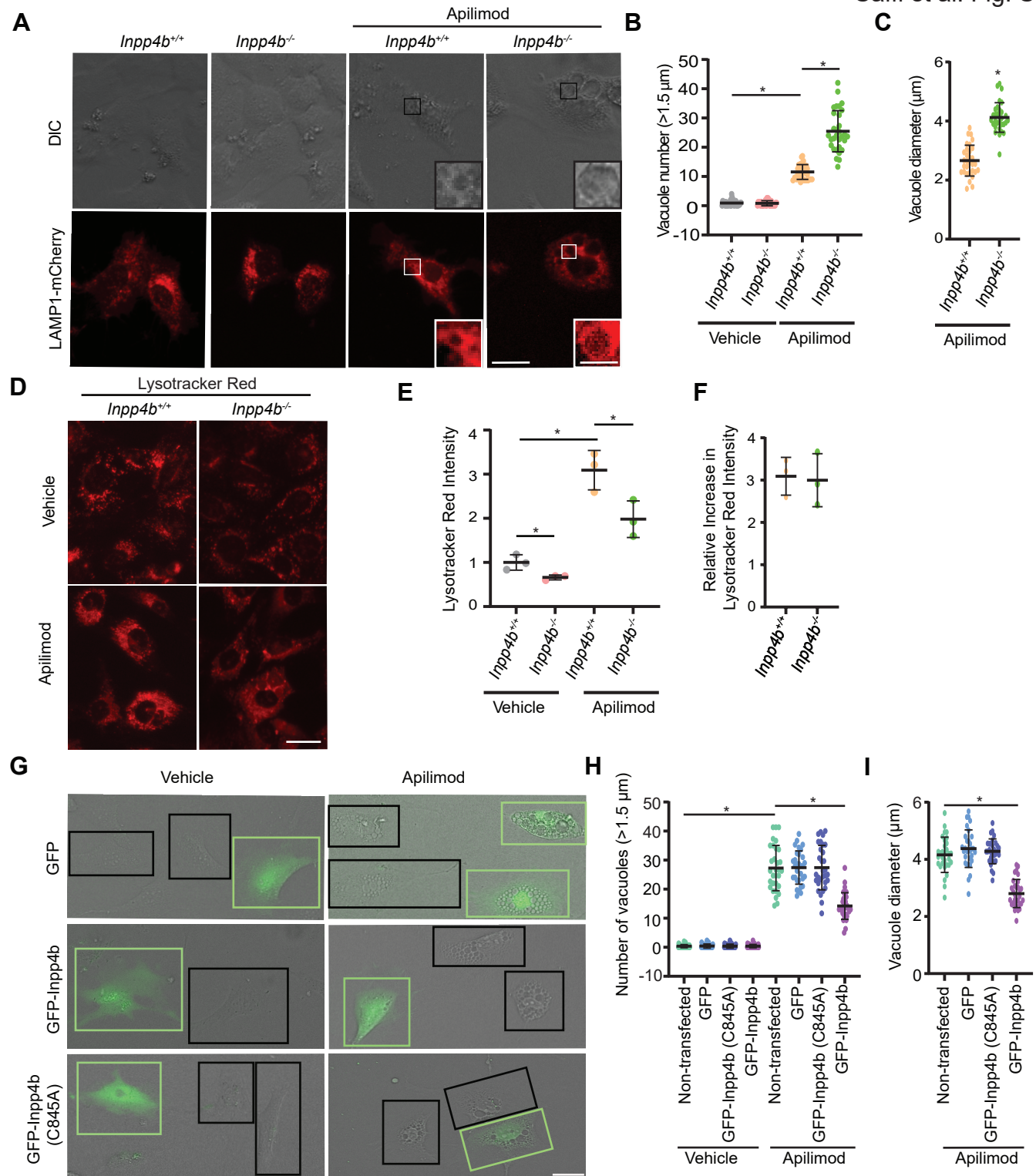

Supplement: Supplementary Figure S1 — Vacuole formation and rescue in Apilimod treated Inpp4b−/−MEFs. (A)Inpp4b+/+ or Inpp4b−/− MEFs transiently expressing LAMP1-mCherry to mark lysosomes followed by vehicle or 10 nM apilimod treatment for 48 h. Quantification of (B) Number of vacuoles (>1.5 μm in diameter) per cell across indicated treatments, and (C) Mean vacuole diameter (μm). Scale Bar: 20 μm, zoomed inset: 5 μm. (D-F)Inpp4b+/+ or Inpp4b−/− MEFs treated with vehicle or apilimod 10 nM for 48 h, followed by Lysotracker Red staining to monitor lysosome levels in Apilimod treated cells. Scale bar: 20 μm. (G)Inpp4b−/− MEFs transiently expressing pEGFP, GFP-Inpp4b, or GFP-Inpp4b (C845A) and treated with vehicle or apilimod 10 nM for 48 h. Transfected cells indicated by green rectangular box outline vs. non-transfected cell(s) indicated by black rectangular box outline. Scale bar: 25 μm. Quantification of (H) Number of vacuoles (>1.5 μm in diameter) per cell across indicated treatments, and (I) Mean vacuole diameter (μm). Data represent ± SD from three independent experiments with 25 to 30 cells assessed per treatment condition per experiment for A-C or 10,000 events per treatment condition per experiment for E. Statistical significance was measured by ANOVA and multiple Student’s t test and represented as ∗ (P < 0.05) [file mmc5.pdf]

**A**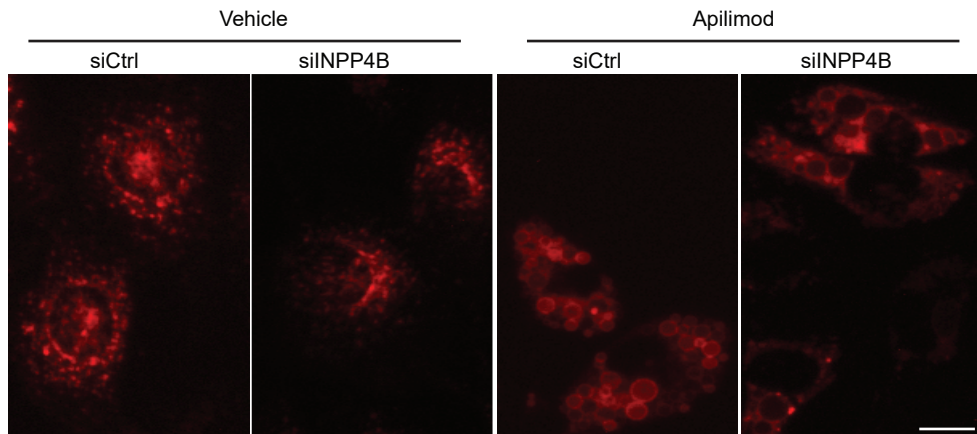**B**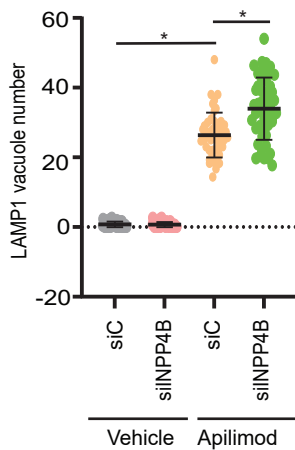**C**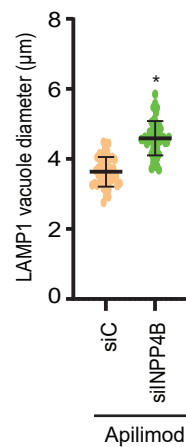**D**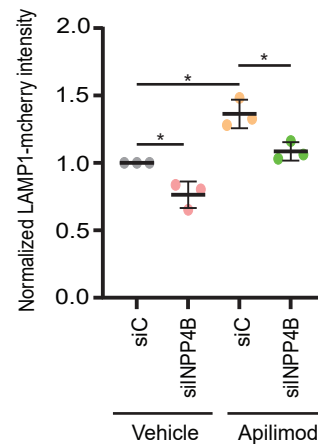**E**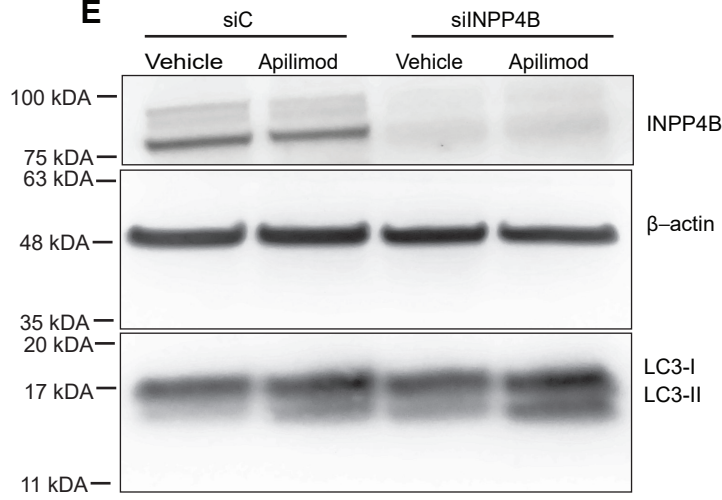

Supplement: Supplementary Figure S2 — Effect of INPP4B and PIKfyve suppression on lysosomes and autophagosomes in U2OS cells. (A) U2OS cells stably expressing mCherry-LAMP1 and treated with vehicle or apilimod 10 nM for 48 h in control (siCtrl) and INPP4B-silenced cells (siINPP4B). (B) Quantification of mCherry-LAMP1 positive vacuole number (>1.5 μm in diameter) per cell across indicated treatments, (C) Mean vacuole diameter (μm) positive for mCherry-LAMP1 per cell across apilimod treated conditions and (D) mCherry-LAMP1 intensity per cell across indicated conditions as measured through flow cytometry. Scale bar: 20 μm. (E) U2OS cells from (A) immunoblotted for INPP4B, LC-3 and beta actin. Data represent ± SD from three independent experiments with 40 to 50 cells assessed per treatment condition per experiment for A-C or 10,000 events per treatment condition per experiment for D. Statistical significance was measured by ANOVA and multiple Student’s t test and represented as ∗ (P < 0.05) [file mmc6.pdf]

**A**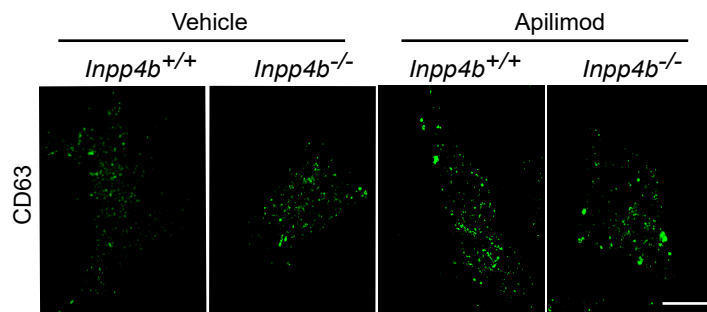**B**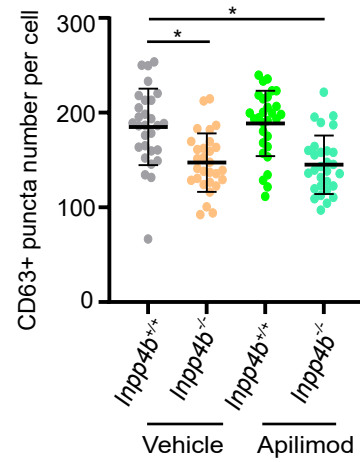

Supplement: Supplementary Figure S3 — Effect of INPP4B and PIKfyve suppression on late endosomes. (A)Inpp4b+/+ or Inpp4b−/− MEFs treated with vehicle or apilimod 10 nM for 48 followed by late endosome immunostaining with CD63 antibody. Scale bar: 20 μm. (B) Quantification of late endosome or CD63+ puncta number per cell across indicated conditions. Data represent ± SD from three independent experiments with 25 to 30 cells assessed per treatment condition per experiment. Statistical significance was measured by ANOVA and multiple Student’s t test and represented as ∗ (P < 0.05) [file mmc7.pdf]

**A**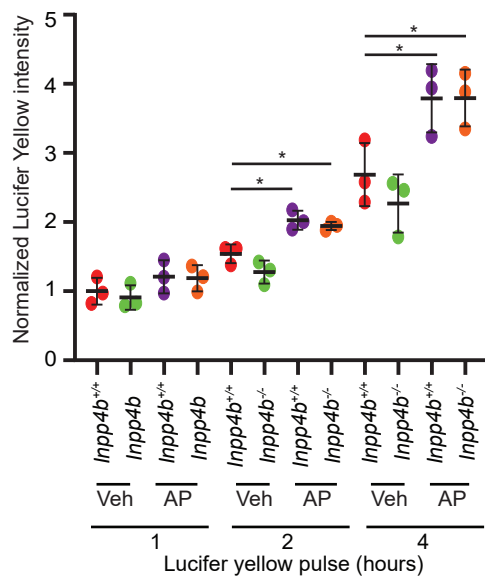**B**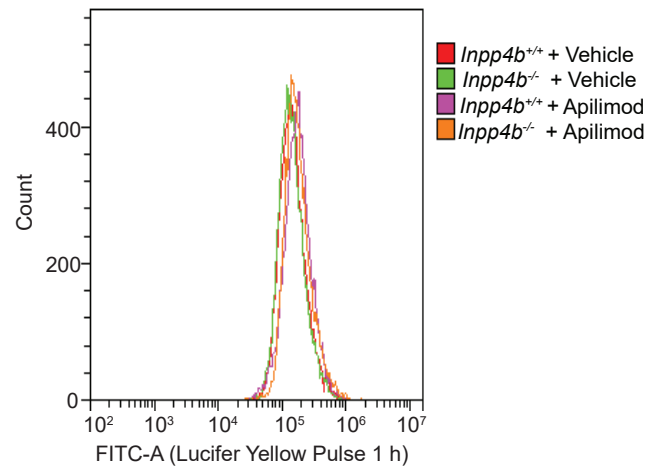**C**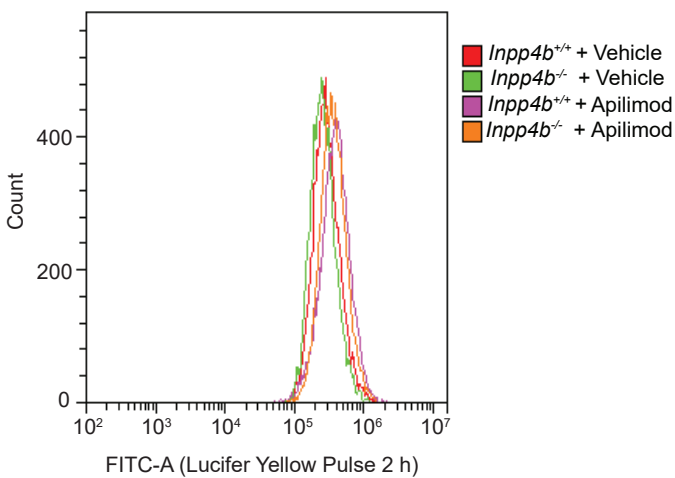**D**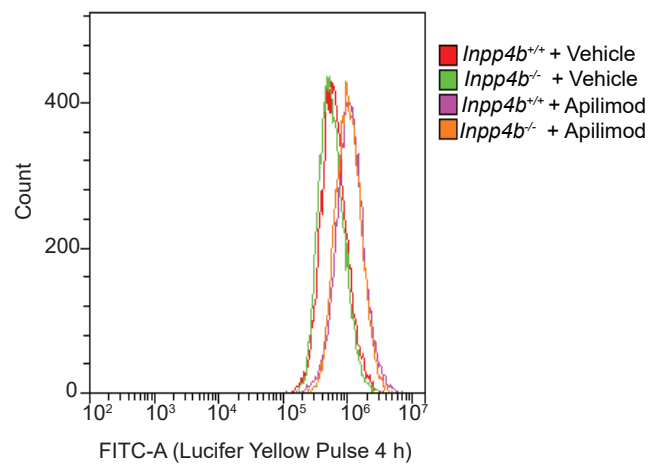

Supplement: Supplementary Figure S4 — Endocytic dynamics of Lucifer Yellow in Inpp4b and PIKfyve suppressed cells. (A)Inpp4b+/+ or Inpp4b−/− MEFs treated with vehicle or apilimod 10 nM for 48 followed by pulsing with Lucifer Yellow for 0, 1, 2 and 4 h. Quantification of Lucifer Yellow intensity as measured through flow cytometry as 10,000 events per treatment condition per experiment. (B-D) Representative flow cytometry plot diagrams of Inpp4b+/+ or Inpp4b−/− MEFs from (A) pulsed with Lucifer Yellow for 1 h or 2 h or 4 h respectively. Statistical significance was measured by ANOVA and multiple Student’s t test and represented as ∗ (P < 0.05) [file mmc8.pdf]

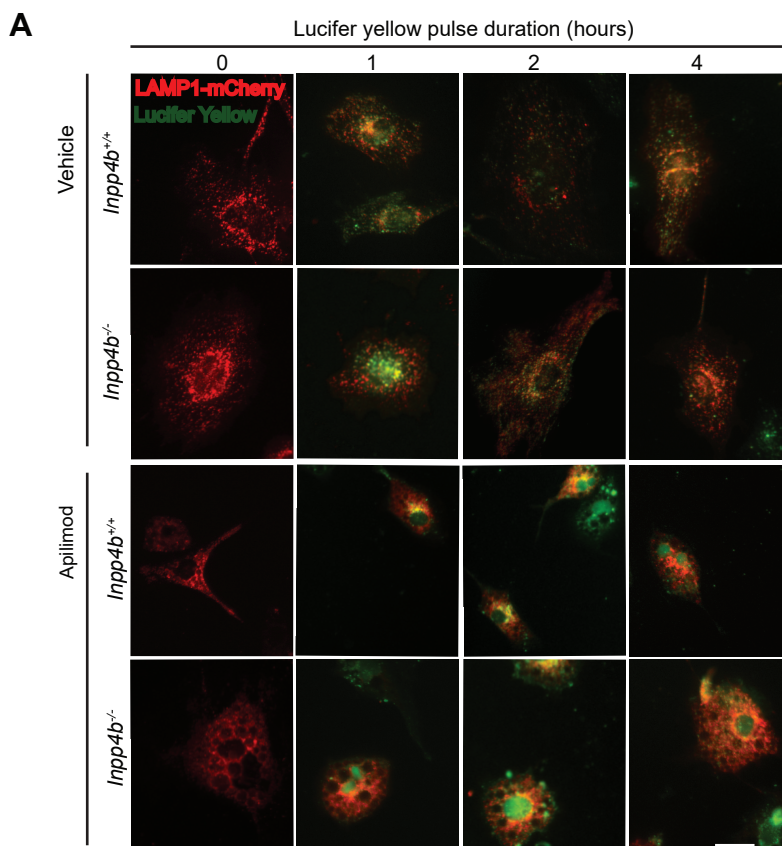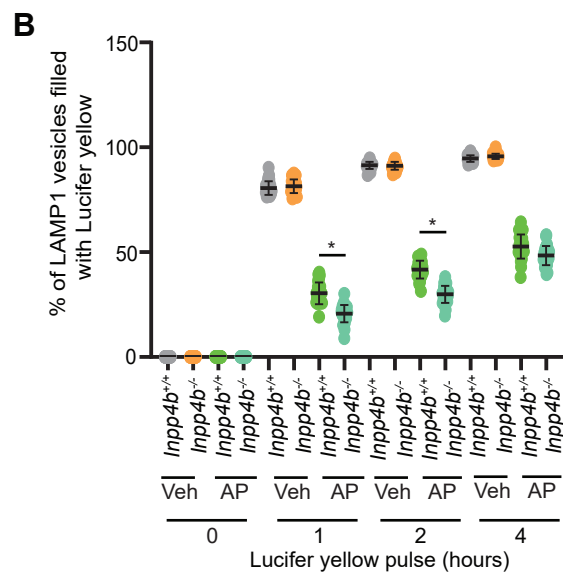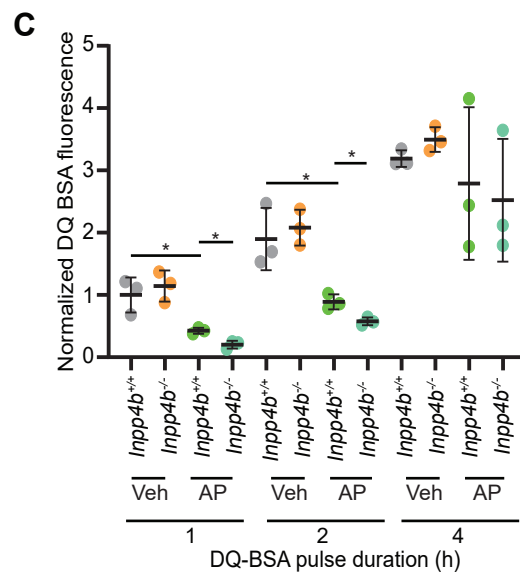

Supplement: Supplementary Figure S5 — Intracellular tracking of endocytic dynamics in Inpp4b and PIKfyve suppressed cells. (A) Inpp4b+/+ or Inpp4b−/− MEFs transiently expressing mCherry-LAMP1 and treated with vehicle or apilimod 10 nM for 48 h. Lucifer yellow pulsed for 0, 1, 2 and 4 h. (B) Quantification of percentage of mCherry-LAMP1 positive vesicles filled with Lucifer Yellow. Scale bar: 25 μm. (C)Inpp4b+/+ or Inpp4b−/− MEFs treated with vehicle or apilimod 10 nM for 48 followed by pulsing with DQ-BSA for 0, 1, 2 and 4 h. Quantification of DQ-BSA intensity as measured through flow cytometry. Data represent + SD from three independent experiments with 25 to 30 cells assessed per treatment condition per experiment for A-B or 10,000 events per treatment condition per experiment for C. Statistical significance was measured by ANOVA and multiple Student’s t test and represented as ∗ (P < 0.05) [file mmc9.pdf]

**A**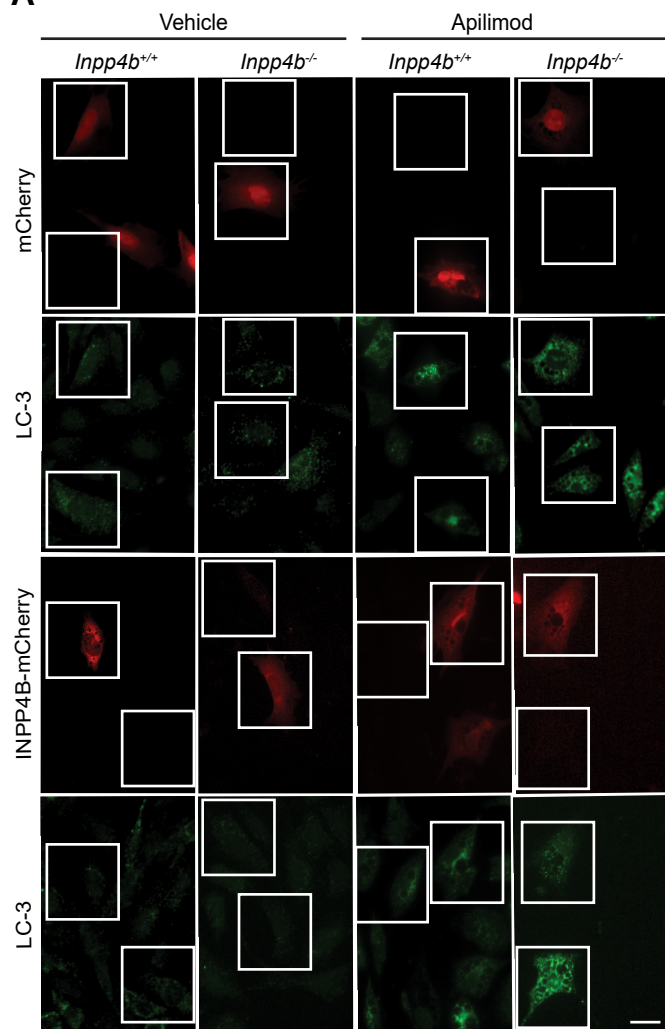**B**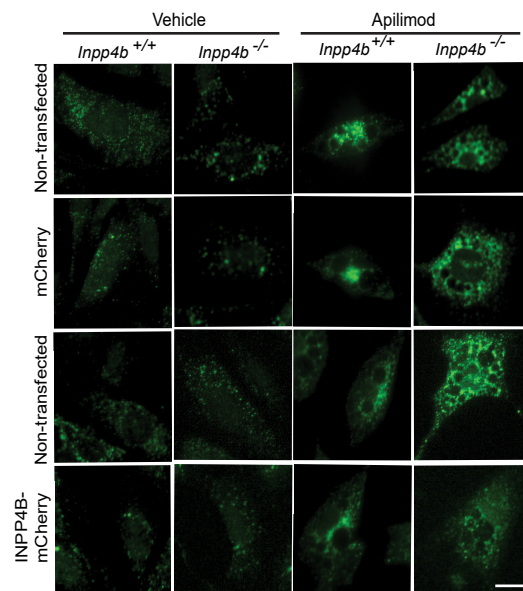**C**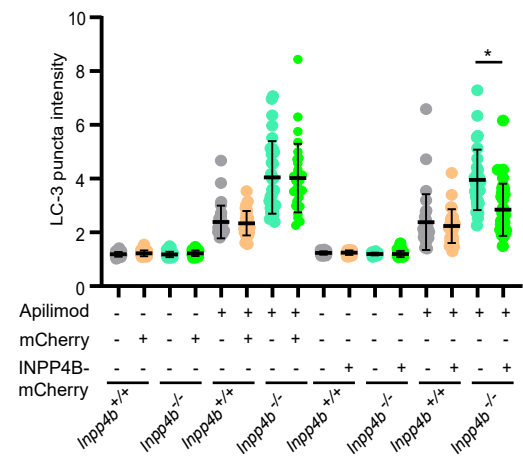

Supplement: Supplementary Figure S6 — INPP4B expression rescue autophagosome defect from PIKfyve suppression. (A)Inpp4b+/+ or Inpp4b−/− MEFs transiently expressing mCherry or INPP4B-mCherry and treated with vehicle or apilimod 10 nM for 48 h. (B) Inset represents a single cell from (A) expanded field of view. (C) Comparison between non-transfected and transfected cells for LC3 puncta intensity per cell across indicated conditions. Scale bar: 10 μm, zoomed inset: 5 μm. Data represent ± SD from three independent experiments with 25 to 30 cells assessed per treatment condition per experiment. Statistical significance was measured by ANOVA and multiple Student’s t test and represented as ∗ (P < 0.05) [file mmc10.pdf]

**A**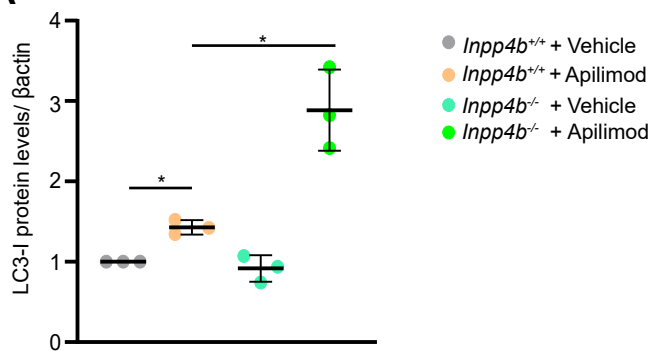**B**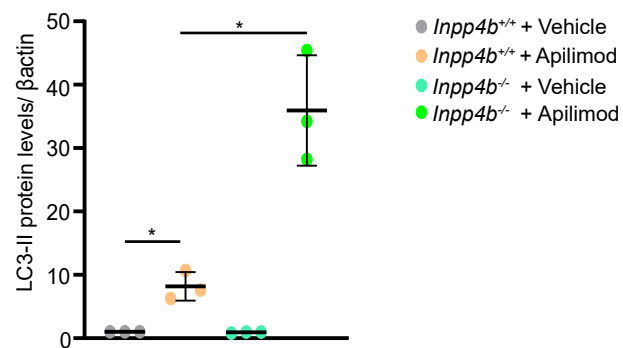**C**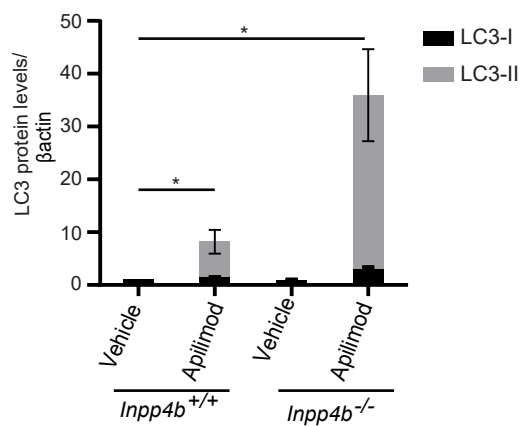

Supplement: Supplementary Figure S7 — Total LC3-I and LC3-II protein levels from Fig. 5D.Inpp4b+/+ or Inpp4b−/− MEFs treated with vehicle or apilimod 10 nM for 48 h, followed by immunoblot and measurement of total (A) LC3-I or (B) LC3-II normalized against Beta-actin, or (C) LC3-I and LC3-II protein levels in a single graph to show relative increase of each protein under indicated treatment conditions. Statistical significance was measured by ANOVA and multiple Student’s t test and represented as ∗ (P < 0.05) [file mmc11.pdf]

**A**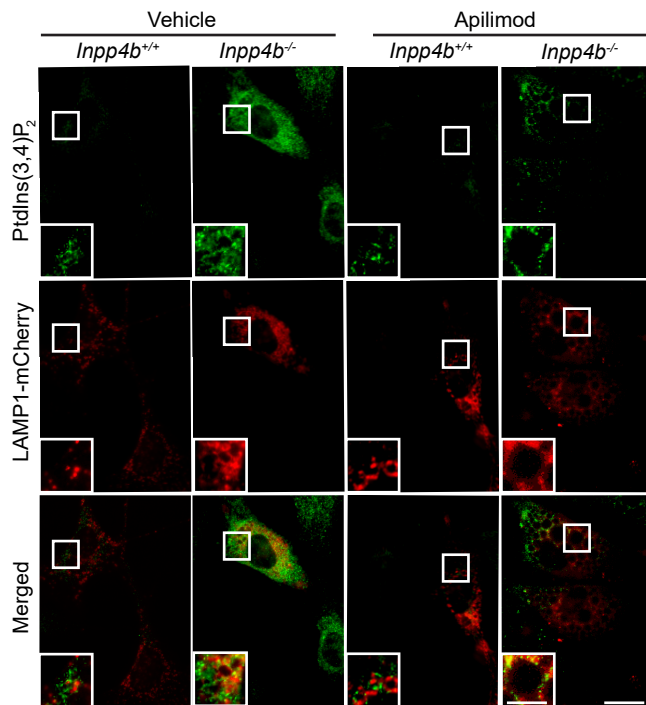**B**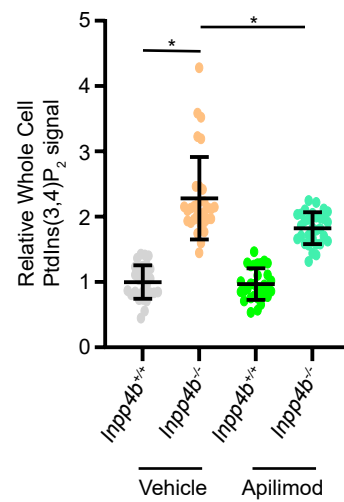**C**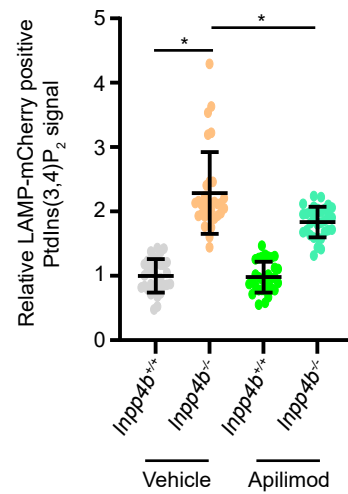

Supplement: Supplementary Figure S8 — PtdIns(3,4)P2phosphoinositide measurements in Inpp4b and PIKfyve suppressed cells. (A)Inpp4b+/+ or Inpp4b−/− MEFs transiently expressing LAMP1-mCherry and treated with vehicle or apilimod 10 nM for 48 h. Quantification of (B) Total cell PtdIns(3,4)P2 or (C) PtdIns(3,4)P 2 fluorescence signal overlayed on LAMP1-mCherry positive regions within a cell. Scale bar: 20 μm, zoomed inset: 5 μm. Data represent ± SD from three independent experiments with 25 to 30 cells assessed per treatment condition per experiment for A-C. Significance measured through multiple Student’s t test represented as ∗ in comparison to indicated conditions (p<0.05) [file mmc12.pdf]
