# Supplementary material for: Inhibition of lipid kinase PIKfyve reveals a role for phosphatase Inpp4b in the regulation of PI(3)P-mediated lysosome dynamics through VPS34 activity
Source: J Biol Chem. 2022 Jun 26;298(8):102187. doi: 10.1016/j.jbc.2022.102187 (PMC9304791; doi:10.1016/j.jbc.2022.102187)

Saffi et al. Fig. S9

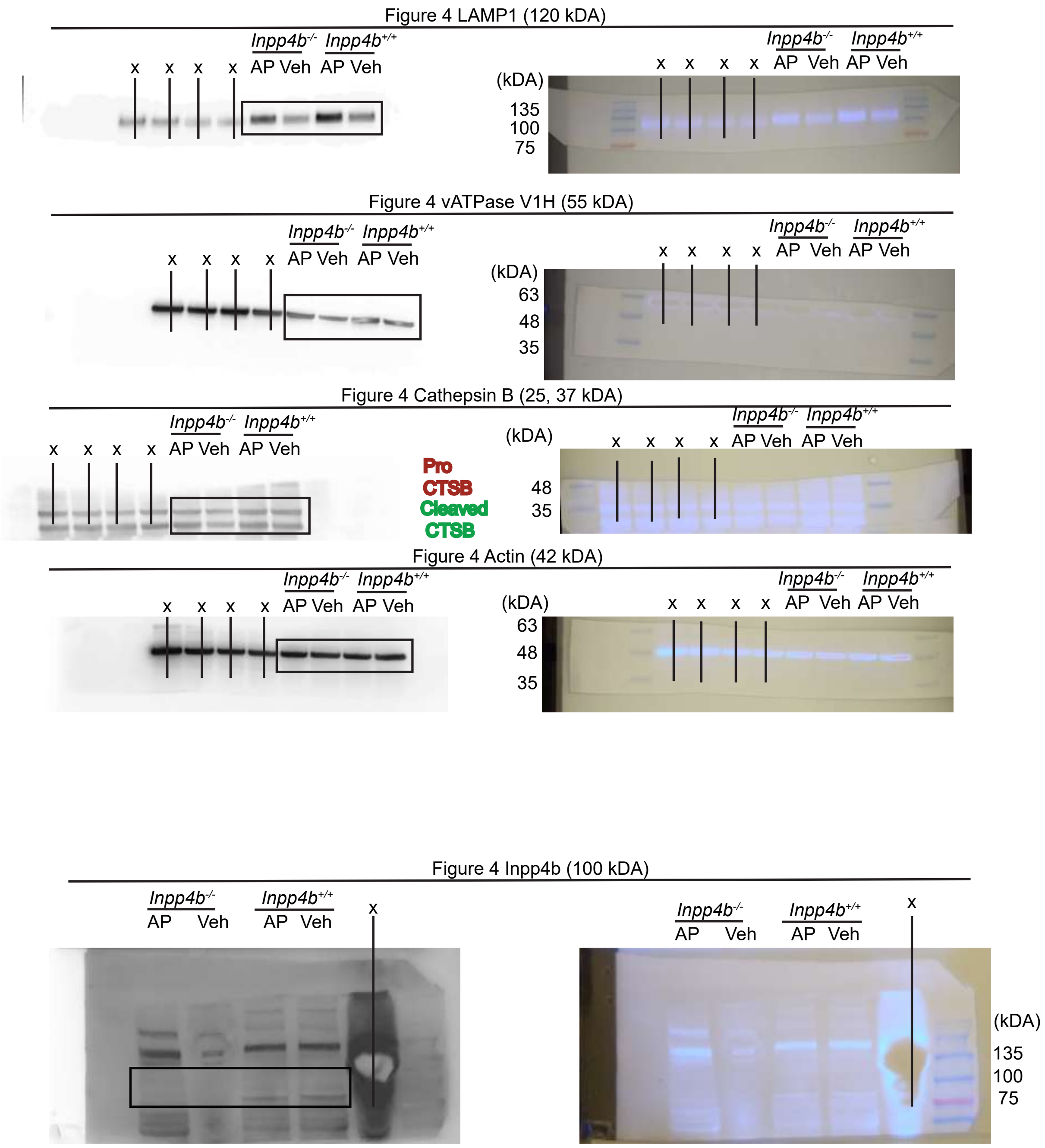

Figure 5 Inpp4b (100 kDA)

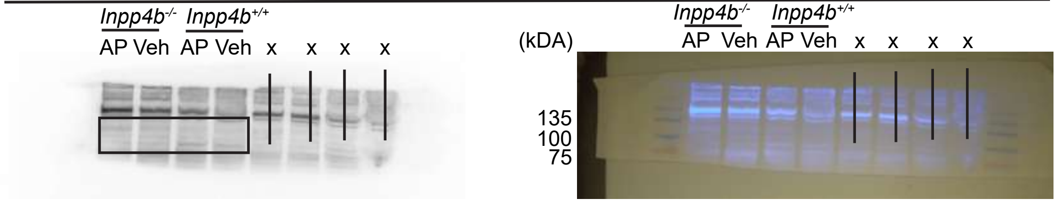


Figure 5 LC3 (14, 16 kDA)

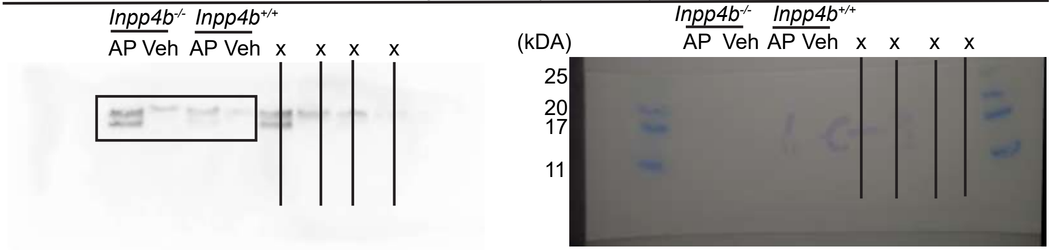


Figure 5 Actin (42 kDA)

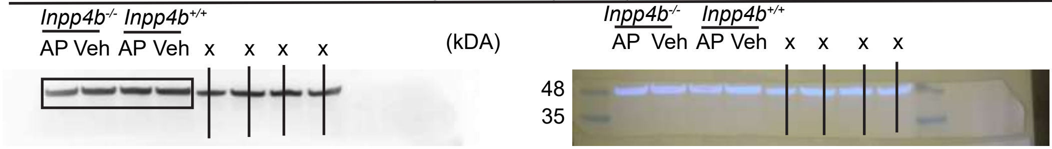

Figure 7 VPS34 (100 kDA)

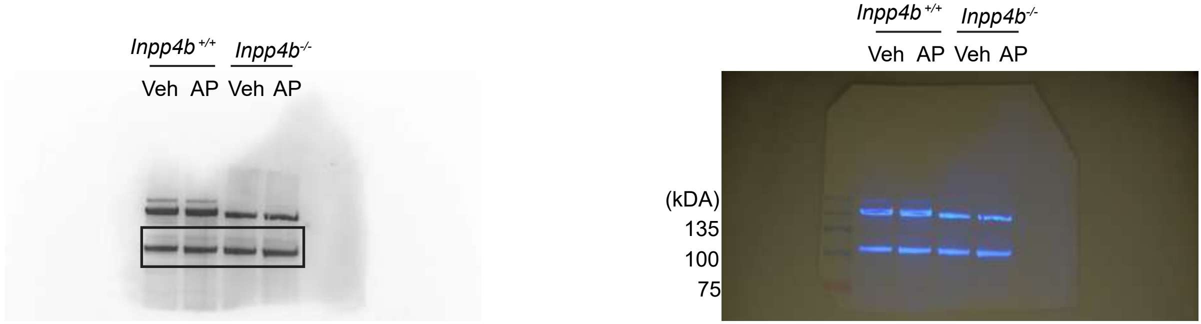


Figure 7 Actin (42 kDA)

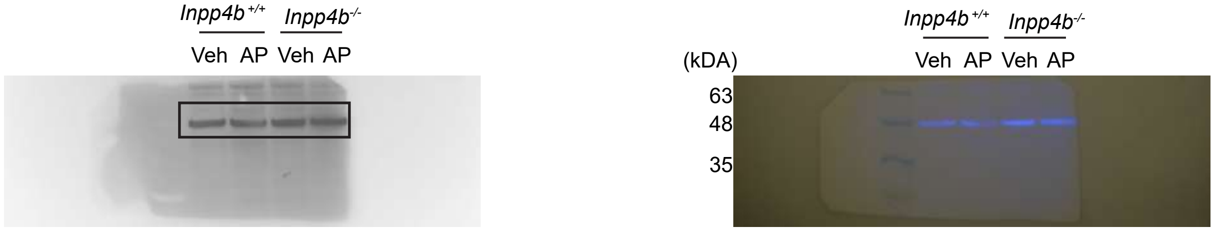


Figure 7 Inpp4b (100 kDA)

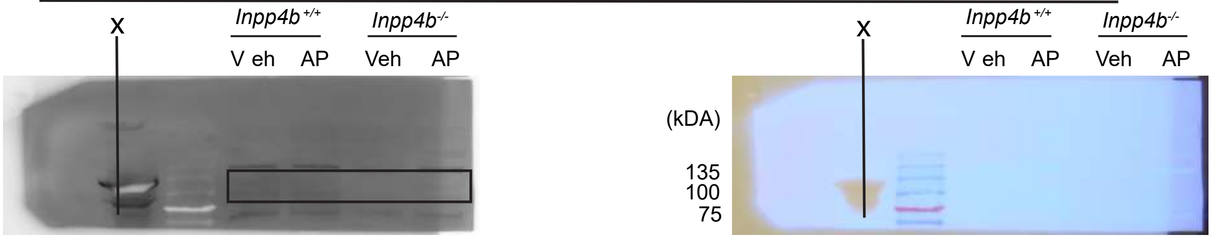


Supplementary Figure 2 INPP4B (100 kDA)

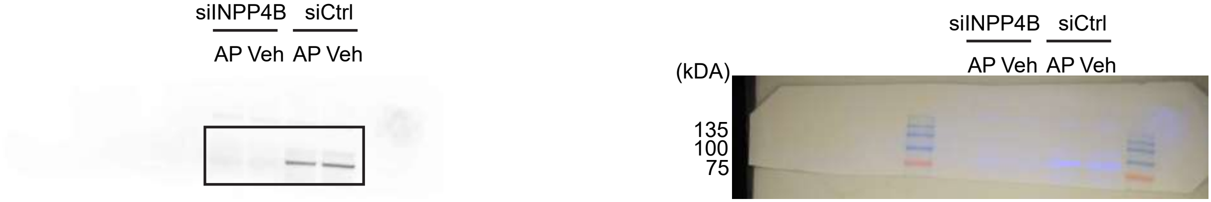


Supplementary Figure 2 Actin (42 kDA)

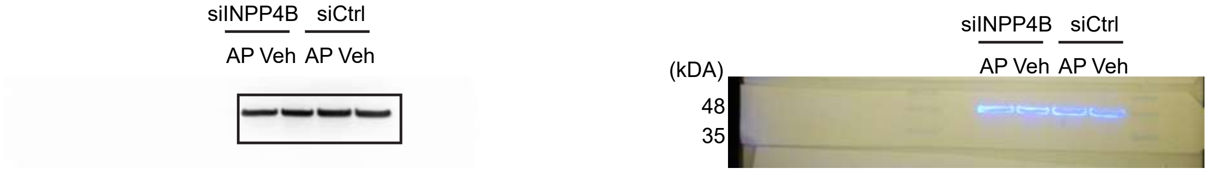


Supplementary Figure 2 LC3 (14, 16 kDA)

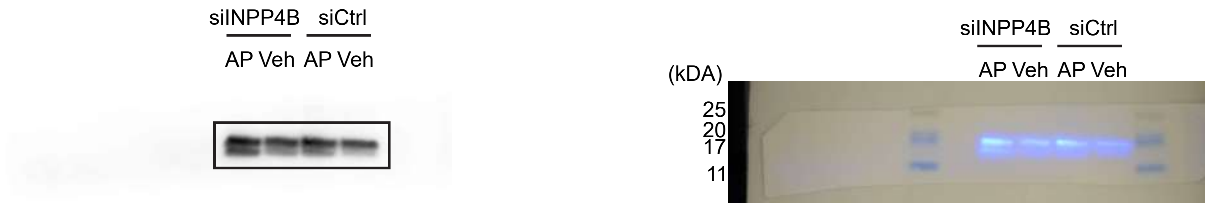

Supplement: Supplementary Figure S9 — Original uncropped western blot images displayed in the manuscript. Complete western blot images with representative ladders and molecular weight markers used to generate manuscript figures. Western blot bands outlined by black box were used for figure generation [file mmc13.pdf]
